# Taming the wild: resolving the gene pools of non-model *Arabidopsis* lineages

**DOI:** 10.1186/s12862-014-0224-x

**Published:** 2014-10-27

**Authors:** Nora Hohmann, Roswitha Schmickl, Tzen-Yuh Chiang, Magdalena Lučanová, Filip Kolář, Karol Marhold, Marcus A Koch

**Affiliations:** Centre for Organismal Studies (COS) Heidelberg, Heidelberg University, Heidelberg, 69120 Germany; Institute of Botany, Academy of Sciences of the Czech Republic, Průhonice, CZ-25243 Czech Republic; Department of Life Sciences, Cheng-Kung University, Tainan, Taiwan; Department of Botany, Faculty of Science, Charles University in Prague, Benátská 2, Prague, CZ-128 01 Czech Republic; Institute of Botany Slovak Academy of Sciences, Dúbravská cesta 9, Bratislava, SK-845 23 Slovakia

**Keywords:** Chloroplast, Cytology, Evolution, ITS, Microsatellites, Systematics, Taxonomy

## Abstract

**Background:**

Wild relatives in the genus *Arabidopsis* are recognized as useful model systems to study traits and evolutionary processes in outcrossing species, which are often difficult or even impossible to investigate in the selfing and annual *Arabidopsis thaliana*. However, *Arabidopsis* as a genus is littered with sub-species and ecotypes which make realizing the potential of these non-model *Arabidopsis* lineages problematic. There are relatively few evolutionary studies which comprehensively characterize the gene pools across all of the *Arabidopsis* supra-groups and hypothesized evolutionary lineages and none include sampling at a world-wide scale. Here we explore the gene pools of these various taxa using various molecular markers and cytological analyses.

**Results:**

Based on ITS, microsatellite, chloroplast and nuclear DNA content data we demonstrate the presence of three major evolutionary groups broadly characterized as *A. lyrata* group, *A. halleri* group and *A. arenosa* group. All are composed of further species and sub-species forming larger aggregates. Depending on the resolution of the marker, a few closely related taxa such as *A. pedemontana*, *A. cebennensis* and *A. croatica* are also clearly distinct evolutionary lineages. ITS sequences and a population-based screen based on microsatellites were highly concordant. The major gene pools identified by ITS sequences were also significantly differentiated by their homoploid nuclear DNA content estimated by flow cytometry. The chloroplast genome provided less resolution than the nuclear data, and it remains unclear whether the extensive haplotype sharing apparent between taxa results from gene flow or incomplete lineage sorting in this relatively young group of species with Pleistocene origins.

**Conclusions:**

Our study provides a comprehensive overview of the genetic variation within and among the various taxa of the genus *Arabidopsis*. The resolved gene pools and evolutionary lineages will set the framework for future comparative studies on genetic diversity. Extensive population-based phylogeographic studies will also be required, however, in particular for *A. arenosa* and their affiliated taxa and cytotypes.

**Electronic supplementary material:**

The online version of this article (doi:10.1186/s12862-014-0224-x) contains supplementary material, which is available to authorized users.

## Background

### *Arabidopsis*: life in the fast lane

Less than a decade ago “*Arabidopsis* and its poorly known relatives” was the title chosen to introduce the closest relatives of *Arabidopsis thaliana* to a broader readership [1]. This review summarized both the systematics and taxonomy of the genus and also the ecologically important traits to be studied in *A. thaliana*’s “wild” relatives. Its necessity was obvious because until 1999, a huge number of species (60) were recognized in *Arabidopsis* in the traditional sense. *Arabidopsis*’ taxonomical history was compiled in detail more than 10 years ago [2,3], and nine *Arabidopsis* species with several subspecies were recognized by this time. Based on this work and unraveling the evolutionary history of the genus *Arabis* [4-7], which differs morphologically from *Arabidopsis* only in the position of the cotyledons relative to the radicle in the seeds, a new systematic concept was presented 10-15 years ago [4,8,9]. Several species and subspecies have since been added either because molecular studies provided new resolution [10] or because description of new species [11] led to changes in their respective taxonomic rank (species, subspecies, variety) [11-17].

*Arabidopsis* has been estimated to comprise of at least nine species and six subspecies [8], or up to 13 (or even more) species and nine subspecies [18] depending on the taxonomic approach and the identifier. The most recent studies, e.g. on *A. arenosa* and its segregates [19], and taxonomic entities within the genus *Arabidopsis* are summarized in Table 1. Note that few of them will probably not be considered in future either because of insufficient diagnostic morphological characters or because they do not represent monophyletic lineages. Russian *Arabidopsis* taxa [17], however, may be considered more carefully in future, based on current morphological and molecular analysis (Koch et al., unpublished data).

**Table 1:** ***Arabidopsis*** **species diversity and taxonomy**

***Arabidopsis arenosa*** **species aggregate**		
*Arabidopsis arenosa* (L.) Lawalrée		
subsp. *arenosa*	(2*n* = 32)	Central and Western Europe, Scandinavia (lower altitudes)
subsp. *arenosa* var. *intermedia* (Kovats) Hayek	(2*n* = 32)	Southeastern Austrian Alps (similar to *A. neglecta*)
subsp. *borbasii* (Zapałowicz) O’Kane & Al-Shehbaz	(2*n* = 32)	Central and Western Europe (mountain ranges, higher altitudes)
*Arabidopsis arenosa*, unclear taxonomic treatment	(2n = 16)	Balkans
*Arabidopsis carpatica*, nom. prov.	(2*n* = 16)	Carpathians (middle altitudes, calcareous bedrocks)
*Arabidopsis neglecta* (Schultes) O’Kane & Al-Shehbaz		
subsp. *neglecta*	(2*n* = 16)	Carpathians (alpine ranges)
subsp. *robusta*, nom. prov.	(2*n* = 32)	Carpathians (alpine ranges, only occasionally in lower altitudes)
*Arabidopsis nitida*, nom. prov.	(2*n* = 16)	Carpathians (mountain ranges, middle to subalpine altitudes)
*Arabidopsis petrogena* (A. Kern) V.I. Dorof.		
subsp. *petrogena*	(2*n* = 16)	Carpathians, Pannonian lowland (maybe two varieties)
subsp. *exoleta*, nom. prov.	(2*n* = 32)	Carpathians (lower altitudes)
***Arabidopsis lyrata*** **lineage**		
*Arabidopsis lyrata* subsp. *lyrata* (L.) O’Kane & Al-Shehbaz	(2*n* = 16)	Alaska, Canada, United States
*Arabidopsis lyrata* subsp. *petraea* (L.) O’Kane & Al-Shehbaz	(2*n* = 16/32)	Europe
*= A. petraea* (L.) V.I. Dorof.		
*Arabidopsis petraea* subsp. *umbrosa* (Turcz. Ex Steud.) Elven & D.F. Murray	(2*n* = 16)	Arctic NE Asia, Siberia, Alaska, Canada
*Arabidopsis petraea* subsp. *septentrionalis* (N. Busch) Elven & D.F. Murray	(2*n* = 32)	Arctic NE Europe, European Russia to Siberia
*Arabidopsis arenicola* (Richardson ex Hook.) Al-Shehbaz et al.	(2*n* = 16)	Arctic Canada and Greenland
***Arabidopsis halleri*** **lineage**		
*Arabidopsis halleri* subsp. *halleri* (L.) O’Kane & Al-Shehbaz	(2*n* = 16)	Europe
*Arabidopsis halleri* subsp. *dacica* (Heuff.) Kolník	(2*n* = 16)	Carpathians, Romania
*Arabidopsis halleri* subsp. *gemmifera* (Matsum.) O’Kane & Al-Shehbaz	(2*n* = 16)	Russia Far East, NE China, Korea, Japan, and Taiwan
*Arabidopsis halleri* subsp. *ovirensis* (Wulfen) A. P. Iljinsk.	(2*n* = 16)	Austria only (all accessions from the Balkans belong to subsp. *halleri*)
*Arabidopsis halleri* subsp. *tatrica* (Pawł.) Kolník	(2*n* = 16)	Tatra mountains, Slovakia
*Arabidopsis umezawana* Kadota	(2*n* = ?)	Japan, Hokkaido (alpine zone of Mt. Rishirizin), annual to biennial
**Other diploid taxa**		
*Arabidopsis pedemontana* (Boiss.) O’Kane & Al-Shehbaz	(2*n* = 16)	NW Italy
*Arabidopsis cebennensis* (DC.) O’Kane & Al-Shehbaz	(2*n* = 16)	SE France, Massif Central
*Arabidopsis croatica* (Schott) O’Kane & Al-Shehbaz	(2*n* = 16)	Croatia
**Allopolyploid taxa**		
*Arabidopsis kamchatica* (Fisch. Ex DC.) O’Kane & Al-Shehbaz	(2*n* = 32)	Boreal Alasca, Canada, E Siberia, Russian Far East, Korea, Japan,
Taiwan		
*Arabidopsis kamchatica* subsp. *kawasakiana* (Makino) Shimizu & Kudoh	(2*n* = 32)	Japan, winterannual (coastal, lowland)
*Arabidopsis suecica* (Fr.) Norrl.	(2*n* = 26)	Fennoscandinavia and the Baltic region

Species diversity of *Arabidopsis thaliana*’s relatives. Information on taxonomy, chromosome number, ploidy level and geographic distribution is provided.

Monophyly is generally accepted among *Arabidopsis* taxa by plant scientists at present. However, considering that *A. thaliana* is a model system taxonomic recognition of new species as *Arabidopsis* is acknowledged much faster than comparable systematic-taxonomic changes in other genera. One such contrary example from the Brassicaceae family is the genus *Noccaea* which includes important model species for heavy metal tolerance and hyperaccumulation. *Noccaea caerulescens* required more than 30 years to be recognized appropriately within the correct evolutionary framework [20,21]. Systematics and taxonomy in the genus *Arabidopsis* is thus ever-debatable and in constant need of further improvement.

### Developing a comprehensive systematic framework

To date there is limited genetic information across the entire genus which allows for adequate taxonomic and systematic comparison. The first study highlighting centers of genetic variation in Europe for the main evolutionary lineages also provided evidence for extensive shared plastidic variation among species [22]. The female component of nuclear-encoded self-incompatibility genes (SI alleles at the SRK locus) also revealed trans-specific polymorphism among some of the same species [23].

Some major evolutionary lineages have been identified in the *Arabidopsis* genus [18,22], namely the following groups: *A. halleri*, *A. lyrata* and *A. arenosa*. Three other genetically isolated diploid species have been identified, *A. croatica*, *A. cebennensis* and *A. pedemontana*. A few allopolyploids are also well studied: *A. suecica* with *A. arenosa* and *A. thaliana* as parental species [24,25], and *A. kamchatica* with *A. lyrata* and *A. halleri* (subsp. *gemmifera*) as parents [26-28]. Another taxonomically not yet introduced tetraploid taxon (close to *A. lyrata*) is found in Lower Austria, which is either the result of hybridization and genome doubling between *A. arenosa* and *A. lyrata* (allopolyploidy), or genome duplication of diploid *A. lyrata* (autopolyploid) with subsequent introgression from tetraploid *A. arenosa* [29].

For some of these major lineages and their subspecies there are more detailed genetic studies available covering either a broader geographic scale or larger sets of taxa. For *A. halleri* it has been shown that all five subspecies are closely related to each other, and that one major center of genetic diversity is located in the Eastern Austrian Alps [22]. It has also been concluded for *A. halleri* that metallicolous populations have been founded separately from distinct non-metallicolous populations without suffering from founder effects [30]. The same authors provided a comprehensive phylogeographic scenario [31]; and although the accessions studied were not characterized taxonomically, many helpful comments linking taxonomy with genetic data were provided. For *A. lyrata* there are several studies available showing general phylogeographic patterns and hybrid speciation on a large scale [26,27]. Local-scale phylogeographic studies in North America highlighted switches in mating system [32,33]. Population-based analysis with a few selected populations provided the first evidence for population genetic structure at varying geographic scales [34-36]. At a more local scale and focusing on different aspects of adaptation there are numerous contributions covering *A. lyrata* [37-39], and comprehensive reviews have recently been presented to summarize many more aspects [1,40]. There is very limited information regarding *A. arenosa*, one of the most diverse evolutionary lineages in *Arabidopsis* [22], with only one phylogeographic-systematic study at a broad geographic scale [19]. Nevertheless, *A. arenosa* has proven to be an excellent model to study the formation and evolution of allopolyploids [41,42] and plant adaptation [43,44].

A recently published review [45] emphasized the need for all-encompassing evolutionary studies within the genus *Arabidopsis* that provide a broader framework on genus-wide genetic diversity and differentiation, in order to enable researchers to study molecular mechanisms of speciation-related processes in interspecific comparative approaches. Our goal here was to provide a reliable phylogenetic-systematic base line using ribosomal DNA sequence variation from the internal transcribed spacers 1 and 2 and the *trn*LF region of the plastid genome [22]. These data were combined with population genetic variation based on a set of nuclear-encoded microsatellite loci shown to be highly sensitive for resolving *Arabidopsis* lineages [29]. Finally, since genome size and chromosome numbers are important cytological characters that significantly influence various organismal traits, we conducted a comprehensive scan of cytological variation via the homoploid nuclear DNA content within and among the principal gene pools in *Arabidopsis.*

Here we explore the gene pools of *Arabidopsis* taxa using a battery of molecular markers and their cytology to identify clearly genetically distinct units over their entire geographic distribution, develop a schematic phylogeographic-systematic scenario based on this data and lastly, comment on any discrepancies between these resolved gene pools and existing taxonomic identifiers.

## Results

**Figure 1:**
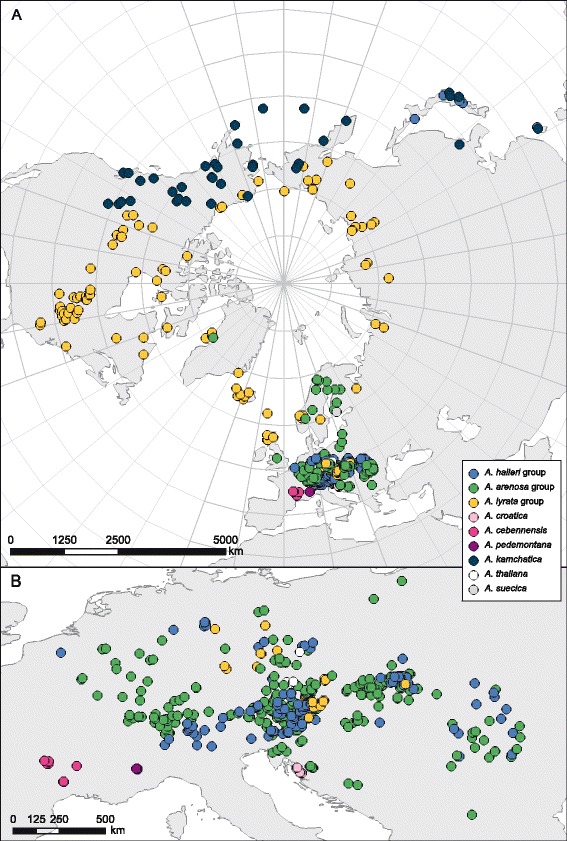
**Sampling distribution.** Geographic distribution of the analyzed *Arabidopsis* taxa. **A)** Circumpolar perspective of sample distribution, **B)** European sampling of accessions. Detailed accession data are provided with Additional file 5.

Our results indicate the existence of several major gene pools or species groups; confirming several taxonomically recognized species and subspecies (Figure 1). However, it is also obvious that gene flow and/or shared ancestry blur some distinct evolutionary units in several cases, both between ploidy levels and among species.

The number of single nucleotide polymorphisms (SNPs) was not sufficient to resolve taxa below the species level, most likely because the genus’ radiation within the last 2.5 million years is too recent.

### ITS sequence data recognize major gene pools

**Figure 2:**
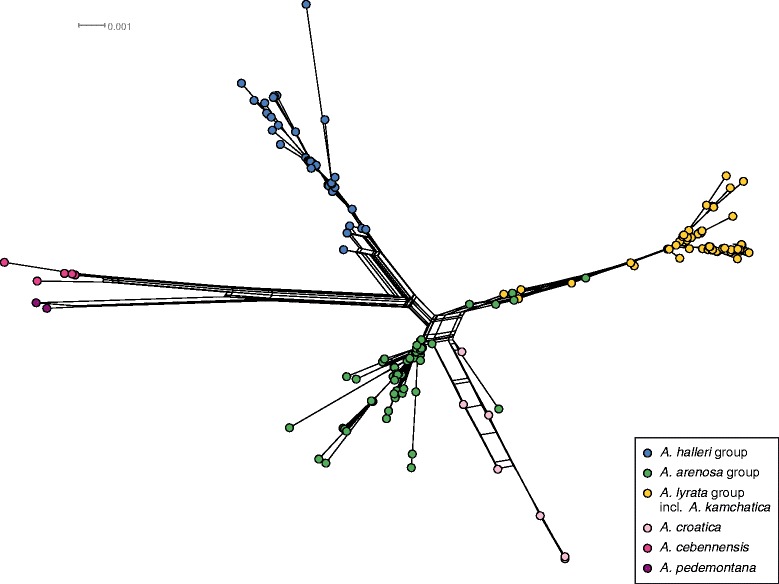
**SplitsTree analysis of ITS data.** Results of the SplitsTree analysis [80] of ITS DNA sequence data. Detailed accession data and ITS type definition are provided with Additional file 5.

The recognition of major gene pools or evolutionary lineages is best illustrated by the SplitsTree analysis based on the ITS (Figure 2). Six major groups with deep splits were detected: 1) *A. halleri* and its subspecies, 2) *A. lyrata* and its segregates and subspecies, including all *A. kamchatica* accessions, 3) *A. arenosa* and its various segregates, subspecies and related taxa (see Tables 1 and 2), 4) diploid *A. croatica*, which is closest to *A. arenosa*, 5) *A. cebennensis*, which is sister to 6) *A. pedemontana*. Notably, the ITS failed to resolve taxa within evolutionary lineages. For example, a few *A. arenosa* accessions cluster within *A. lyrata* or *A. croatica* (one accession). This is best explained by interploidy and interspecies gene flow and/or shared ancestry, as commented on earlier [18,19]. All analyzed *A. suecica* accessions carried ITS types, which clustered with *A. thaliana* ITS types (results not shown). The complete alignment can be viewed in Additional file 1.

**Table 2:** **Estimation of absolute genome size data of** ***Arabidopsis*** **taxa**

	**N**	**1Cx-value (pg) (SD)**	**Expected genome size**	**Calculated genome size** ^**1**^
*Arabidopsis thaliana* (Columbia) (2×)	4	0.173 (0.010)	135 Mbp^(1)^	^(1)^set as reference
*Arabidopsis croatica* (2×)	2	0.225 (0.007)		176 Mbp
*Arabidopsis arenicola* (2×)	4	0.260 (0.008)		203 Mbp
*Arabidopsis lyrata* (2×) (Europe)	4	0.270 (0.005)		210 Mbp
*Arabidopsis lyrata* (2×) (North America)	8	0.274 (0.007)	207 Mbp^(2)^	214 Mbp
*Arabidopsis lyrata* (4×)	4	0.238 (0.005)		371 Mbp
*Arabidopsis halleri* ssp. *halleri* (2×)	10	0.266 (0.010)		207 Mbp
*Arabidopsis halleri* ssp. *gemmifera* (2×)	6	0.270 (0.004)		211 Mbp
*Arabidopsis kamchatica* (4×)	2	0.235 (0.007)		367 Mbp
*Arabidopsis arenosa* (4×)	6	0.237 (0.005)		370 Mbp
*Arabidopsis cebennensis* (2×)	22	0.281 (0.010)		219 Mbp
*Arabidopsis pedemontana* (2×)	6	0.277 (0.005)		216 Mbp

Genome sizes measured as 1Cx-values in pg. *Arabidopsis thaliana* was used as absolute reference of genome size (^1^135 Mbp, TAIR: http://www.arabidopsis.org) with standard deviation (SD) provided. All genome size data were extrapolated as haploid genome size (1C) accordingly to achieve absolute genome sizes in Mbp. *Arabidopsis lyrata* from North America served as second and independent control since its genome has been also sequenced and fully annotated ^2^(207 Mbp [46]). Ploidy level is given in brackets after the respective taxon name. (N: number of individuals analyzed).

### Nuclear DNA content supports the distinction of major ITS gene pools

**Figure 3:**
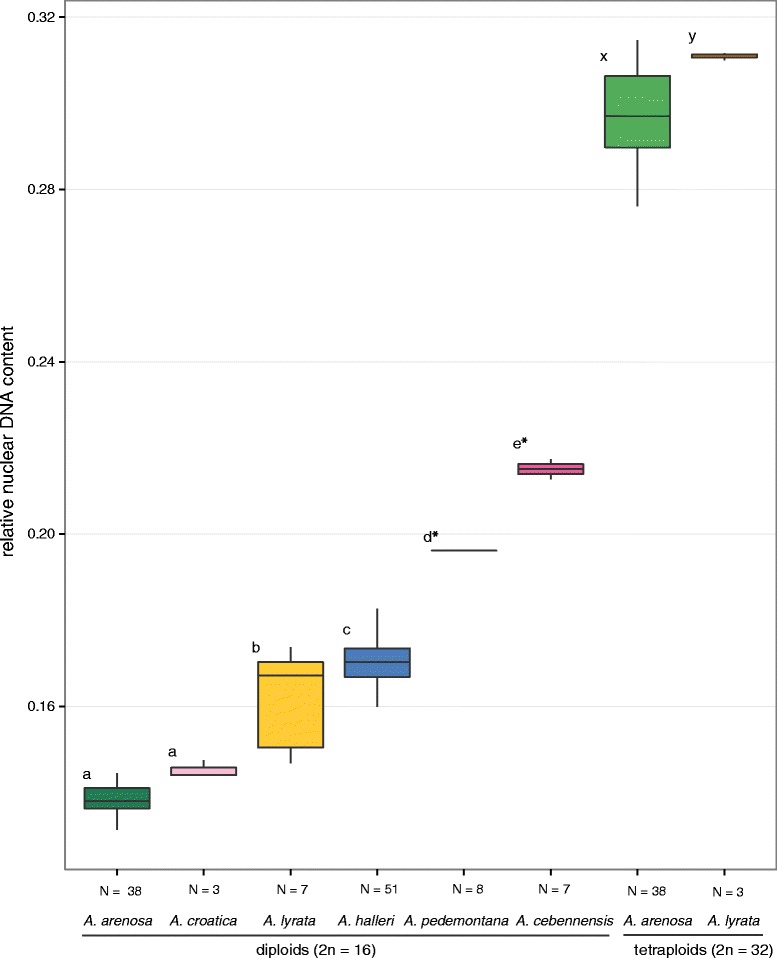
**DNA content variation in the genus** ***Arabidopsis.*** Variation in nuclear DNA contents (reference standard set as 1) among major gene pools of *Arabidopsis* (excluding *A. thaliana* and hybridogenous taxa) determined by flow cytometry of 143 accessions from throughout Europe and Japan. Fluorescence intensity of *Solanum pseudocapsicum* was set to a unit value. Letters indicate significantly different groups at α = 0.05 as indicated by TukeyHSD post-hoc multiple comparison test (diploid and tetraploid accessions were tested separately; *were marginally significant at p = 0.055). The values represented by lines, boxes and whiskers are median, quartiles and range (min-max), respectively.

The major gene pools identified by ITS sequences were also significantly differentiated by their homoploid nuclear DNA content (Figure 3). Disregarding *A. thaliana,* with a basic chromosome number of n = 5 (1C value of about 0.17 pg) and on average 47% less DNA than the other diploid accessions, the homoploid nuclear DNA content varied 1.66–fold among diploid and 1.14–fold among tetraploid accessions, respectively. The differences among major gene pools were highly significant among diploid (F_5,96_ = 212, p <0.0001) and marginally significant among tetraploid (F_1,39_ = 4.8, p = 0.03) accessions. At the diploid level, accessions of *A. arenosa* possessed the lowest nuclear DNA content, followed by *A. croatica* (5% larger DNA content than *A. arenosa*, but not significant), *A. lyrata* (17%), *A. halleri* (23%) and, finally, *A. pedemontana* (42%) and *A. cebennensis* (55% larger DNA content than *A. arenosa*; Figure 3). Interestingly, *A. croatica* and *A. arenosa* was the only species pair with non-significant differentiation in nuclear DNA contents. Among tetraploids, *A. lyrata* exhibited on average 5% lower nuclear DNA content than *A. arenosa*, although it still fell within the range of *A. arenosa* variation.

In contrast to among-group nuclear DNA content, variation was markedly reduced within the major gene pools and differences among accessions were minimal (the DNA content varied 1.18–fold, 1.1–fold and 1.14–fold, within diploid *A. lyrata*, *A. arenosa*, and *A. halleri* gene pools, respectively; and 1.01–fold and 1.14–fold within tetraploid *A. lyrata* and *A. arenosa*, respectively). In tetraploids, the variation was 1.01–fold and 1.14–fold among *A. lyrata* and *A. arenosa* accessions, respectively.

Absolute genome size estimates are also provided for all taxa in Table 2 in a Brassicaceae-wide screen. The 1C-value of *A. thaliana* ecotype Columbia is about 0.17 pg. The estimated physical size of its genome is currently about 135 Mbp, (TAIR, http://www.arabidopsis.org/). Our estimated 1C-value of 0.274 pg for North American *A. lyrata* indicates a respective genome size of approximately 214 Mbp and is very close to the published physical size (207 Mbp) of the *A. lyrata* genome [46]. The discrepancy of about 5% could be explained by missing sequence data from centromeric regions.

### Chloroplast sequence data recognize some major gene pools but indicates shared polymorphism

**Figure 4:**
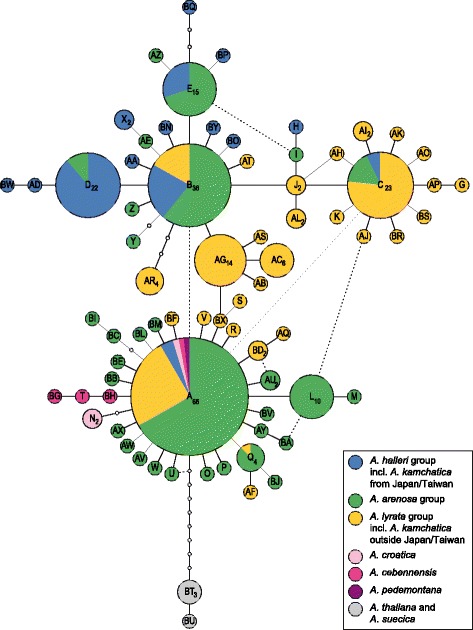
**Chloroplast DNA type network analysis in the genus** ***Arabidopsis.*** The chloroplast DNA (*trn*LF) network from the TCS analysis [76] recognized 71 cpDNA suprahaplotypes and two additional suprahaplotypes from *A. thaliana*/*A. suecica.* The size of the circle represents the number of sequence types within the suprahaplotype. Dash lines indicate not significant connections as revealed with maximum-likelihood tree building analysis. Detailed accession data are provided with Additional file 5.

In contrast to the ITS, plastid *trnLF* sequences did not fully resolve all evolutionary lineages. The TCS network recognized 71 suprahaplotypes and two additional suprahaplotypes from *A. thaliana*/*A. suecica* (Figure 4). Central suprahaplotypes in the network with the highest frequency of occurrence (A, B, C, D, E) were largely shared among lineages (as defined by ITS). In agreement with placement of the root (*A. thaliana*), haplotype A was the most ancestral (occurring also with the highest frequency), and it was shared among all lineages. Suprahaplotypes B and C were shared among the three major lineages (*A. lyrata*, *A. arenosa*, and *A. halleri*), and suprahaplotypes D and E were shared by *A. halleri* and *A. arenosa* only. Insufficient resolution in the chloroplast suggests the presence of shared ancestral gene pools and subsequent incomplete lineage sorting [29]; and/or hybridization and introgression which in some cases resulted in stable allopolyploids (e.g. *A. kamchatica*, *A. suecica*). In particular, hybridization and introgression may not be resolved by ITS data because of rapid and ongoing concerted evolution [27,47]. Past interploidy and interspecific gene flow has been demonstrated among European *Arabidopsis* species [48], and introgression zones can indeed have larger geographic extension and long-term persistence [29]. One notable detail taken from the TCS network is that connecting haplotypes were rarely missing. This might be anindicator for (overall) limited bottlenecks and large past effective population sizes [49]. The *trnLF* alignment is shown in Additional file 2, and a summary of all suprahaplotypes and their distribution among taxa is shown in Additional file 3.

### Microsatellite analyses characterize distinctive taxa and cytotypes

A summary statistics table for microsatellite alleles within the various taxa is given with Table 3, and displays total number of alleles, mean number of alleles per locus, number of unique alleles, and number of rare alleles (<5%). These data show that tetraploids have a significantly higher number of alleles per locus per individual (normally exceeding 2 alleles per locus) (p < 0.001) than diploids, as one would expect. The highest numbers of total alleles were found within widely distributed diploid *A. lyrata* subsp. *petraea*, tetraploid *A. arenosa* subsp. *arenosa* and subsp. *borbasii*, but also with diploid *A. carpatica* and tetraploid *A. petrogena* subsp. *exoleta*, which highlights the importance of the *A. arenosa* gene pool as highly diverse [22]. Accordingly, the same taxa did not only carry the highest numbers of unique alleles but also rare alleles (frequency 5% and lower in the whole dataset). It is also demonstrated by the summary statistics that local endemics such as *A. pedemontana*, *A. cebennensis*, or *A. croatica* (all of them endangered and highly protected) had much lower numbers for any genetic scoring value (Tables 3 and 4). This was also true for North American *A. lyrata* subsp. *lyrata* and *A. arenicola*.

**Table 3:** ***Arabidopsis*** **microsatellite genetic variation**

**Taxon**	**Ploidy**	**Mating system**	**Individuals (populations)**	**No. of alleles**	**Mean no. of alleles per locus (SD)** ^**2**^	**No. of unique alleles**	**No. of rare alleles (5%)**
*A. arenicola*	Diploid	SC	19 (14)	17	1.04 (0.10)	-	-
*A. lyrata* subsp. *lyrata*	Diploid	SI/SC^1^	57 (29)	38	1.18 (0.16)	-	-
*A. petraea* susbp. *umbrosa*	Diploid	Unknown	25 (16)	29	1.20 (0.26)	-	2
*A. petraea* subsp. *septentrionalis*	Tetraploid	Unknown	8 (8)	36	1.63 (0.31)	2	2
*A. lyrata* subsp. *petraea*	Diploid	SI	187 (13)	89	1.46 (0.19)	10	13
*A. lyrata* subsp. *petraea*	Tetraploid	SI	25 (5)	57	1.93 (0.37)	-	-
*A. arenosa* subsp. *arenosa*	Tetraploid	SI	76 (13)	94	2.31 (0.33)	2	5
*A. arenosa* subsp*. arenosa* var*. intermedia*	Tetraploid	SI	14 (4)	44	2.19 (0.27)	-	-
*A. arenosa* subsp. *borbasii*	Tetraploid	SI	160 (22)	111	2.30 (0.33)	7	9
*A. carpatica*	Diploid	SI	113 (9)	104	1.59 (0.19)	6	10
*A. petrogena* subsp. *petrogena*	Diploid	SI	73 (7)	73	1.53 (0.20)	-	3
*A. petrogena* subsp. *exoleta*	Tetraploid	SI	56 (11)	83	2.22 (0.36)	1	5
*A. neglecta* subsp. *neglecta*	Diploid	SI	35 (5)	51	1.51 (0.21)	1	3
*A. neglecta* subsp. *robusta*	Tetraploid	SI	8 (1)	39	2.24 (0.23)	-	2
*A. nitida*	Diploid	SI	22 (4)	59	1.54 (0.22)	3	3
*A. croatica*	Diploid	SI	15 (3)	26	1.33 (0.19)	1	1
*A. halleri* subsp. *dacica*	Diploid	SI	3 (3)	15	1.53 (0.31)	-	-
*A. halleri* subsp. *halleri*	Diploid	SI	199 (19)	59	1.37 (0.19)	6	7
*A. halleri* subsp*. ovirensis*	Diploid	SI	24 (4)	18	1.31 (0.16)	1	1
*A. halleri* subsp. *tatrica*	Diploid	SI	25 (7)	40	1.37 (0.14)	-	1
*A. halleri* subsp. *gemmifera*	Diploid	SI	8 (5)	15	1.10 (0.15)	-	-
*A. cebennensis*	Diploid	SI	153 (11)	23	1.17 (0.14)	-	-
*A. pedemontana*	Diploid	SI	40 (9)	22	1.31 (0.18)	-	1

Summary table of analyzed individuals and populations of the various *Arabidopsis* taxa for microsatellite variation. Information on ploidy level, mating system variation (own data and literature survey) and some summary statistics are provided. SC: self-compatible, SI: self-incompatible.

^1^SC populations of North American *A. lyrata* subsp. *lyrata* are not considered here.

^2^Respectively two loci in tetraploids.

**Table 4:** **Gene diversity statistics of microsatellites, ITS and cpDNA variation**

		**Microsatellites**		**ITS haplotypes**			***trn*** **LF haplotypes**		
	**Ploidy**	**Individuals analyzed**	**Nei’s gene diversity (SD)**	**Individuals analyzed**	**Nucleotide diversity (πx10** ^**−2**^ **) (SD)**	**Nei’s gene diversity (SD)**	**Individuals analyzed**	**Nucleotide diversity (πx10** ^**−2**^ **) (SD)**	**Nei’s gene diversity (SD)**
***A. lyrata*** **group**		**288 (85)**	**0.562 (0.312)**	**126 (92)**	**0.248 (0.168)**	**0.797 (0.025)**	**176 (101)**	**0.224 (0.150)**	**0.751 (0.024)**
*A. arenicola*	2×	19 (14)	0.205 (0.156)	17 (16)	0.139 (0.117)	0.323 (0.135)	17 (15)	0.035 (0.047)	0.117 (0.101)
*A. lyrata* subsp. *lyrata*	2×	57 (29)	0.378 (0.225)	37 (21)	0.014 (0.030)	0.516 (0.085)	54 (26)	0.058 (0.061)	0.380 (0.065)
*A. petraea* subsp. *umbrosa*	2×	25 (16)	0.468 (0.272)	30 (18)	0.154 (0.118)	0.675 (0.061)	28 (18)	0.362 (0.225)	0.738 (0.053)
*A. petraea* subsp. *septentrionalis*	4×	8 (8)	not calculated	10 (10)	0.030 (0.046)	0.200 (0.154)	9 (9)	0.323 (0.223)	0.694 (0.147)
*A. lyrata* subsp. *petraea*	2×	187 (13)	0.556 (0.309)	31 (26)	0.025 (0.039)	0.898 (0.030)	61 (29)	0.229 (0.154)	0.766 (0.042)
*A. lyrata* subsp. *petraea*	4×	25 (5)	not calculated	1 (1)	0.000	1.000 (0.000)	7 (4)	0.241 (0.185)	0.714 (0.180)
***A. arenosa*** **group**		**258 (79)**	**0.560 (0.311)**	**247 (181)**	**0.138 (0.107)**	**0.803 (0.024)**	**568 (263)**	**0.171 (0.123)**	**0.585 (0.023)**
*A. arenosa* subsp. *arenosa*	4×	76 (13)	not calculated	23 (23)	0.129 (0.106)	0.806 (0.061)	32 (28)	0.163 (0.122)	0.485 (0.107)
*A. arenosa* var. *intermedia*	4×	14 (4)	not calculated	4 (3)	0.000	1.000 (0.176)	6 (5)	0.149 (0.133)	0.333 (0.215)
*A. arenosa* subsp. *borbasii*	4×	160 (22)	not calculated	173 (120)	0.065 (0.066)	0.748 (0.033)	391 (167)	0.142 (0.107)	0.503 (0.029)
*A. carpatica*	2×	113 (9)	0.554 (0.309)	8 (6)	0.065 (0.075)	0.750 (0.139)	51 (14)	0.282 (0.181)	0.752 (0.046)
*A. petrogena*	2×	73 (7)	0.512 (0.289)	5 (4)	0.399 (0.298)	1.000 (0.298)	37 (20)	0.052 (0.058)	0.348 (0.077)
*A. petrogena* subsp. *exoleta*	4×	56 (11)	not calculated	8 (6)	0.345 (0.241)	0.928 (0.084)	8 (6)	0.341 (0.238)	0.892 (0.085)
*A. neglecta* subsp. *neglecta*	2×	35 (5)	0.451 (0.262)	8 (5)	0.323 (0.229)	0.928 (0.084)	16 (5)	0.111 (0.097)	0.450 (0.150)
*A. neglecta* subsp. *robusta*	4×	8 (1)	not calculated	6 (3)	0.000	0.600 (0.215)	6 (3)	0.099 (0.101)	0.333 (0.215)
*A. nitida*	2×	22 (4)	0.635 (0.354)	4 (4)	0.384 (0.309)	1.000 (0.176)	11 (6)	0.282 (0.196)	0.709 (0.099)
*A. croatica*	2×	15 (3)	0.374 (0.228)	8 (7)	0.384 (0.263)	1.000 (0.062)	10 (9)	0.159 (0.129)	0.533 (0.094)
***A. halleri*** **group**		**259 (38)**	**0.427 (0.254)**	**103 (90)**	**0.159 (0.118)**	**0.901 (0.020)**	**94 (83)**	**0.268 (0.173)**	**0.712 (0.030)**
*A. halleri* subsp. *dacica*	2×	3 (3)	0.533 (0.380)	8 (7)	0.049 (0.063)	0.928 (0.084)	8 (7)	0.213 (0.165)	0.464 (0.200)
*A. halleri* subsp. *halleri*	2×	199 (19)	0.392 (0.237)	67 (61)	0.036 (0.047)	0.858 (0.029)	62 (58)	0.236 (0.158)	0.670 (0.049)
*A. halleri* subsp. *ovirensis*	2×	24 (4)	0.330 (0.204)	5 (4)	0.122 (0.122)	0.900 (0.161)	2 (1)	0.000	0.000
*A. halleri* subsp. *tatrica*	2×	25 (7)	0.408 (0.242)	13 (10)	0.023 (0.039)	0.730 (0.096)	12 (10)	0.364 (0.238)	0.727 (0.113)
*A. halleri* subsp. *gemmifera*	2×	8 (5)	0.301 (0.205)	9 (7)	0.570 (0.363)	0.916 (0.092)	9 (6)	0.000	0.000
*A. umezawana*	?	0	not calculated	1 (1)	0.000	1.000 (0.000)	1 (1)	0.000	1.000 (0.000)
***A. cebennensis***	**2×**	**153 (11)**	**0.189 (0.130)**	**8 (6)**	**0.185 (0.150)**	**0.785 (0.150)**	**148 (12)**	**0.174 (0.125)**	**0.702 (0.018)**
***A. pedemontana***	**2×**	**40 (9)**	**0.259 (0.167)**	**2 (2)**	**0.312 (0.382)**	**1.000 (0.500)**	**9 (2)**	**0.000**	**0.000**

Nei’s Gene and nucleotide diversity [89] of microsatellite, ITS and cpDNA genetic variation among the various taxa. The number of individuals analyzed is indicated with respective number of populations in brackets. Standard deviation of mean genetic diversity is given in brackets.

**Figure 5:**
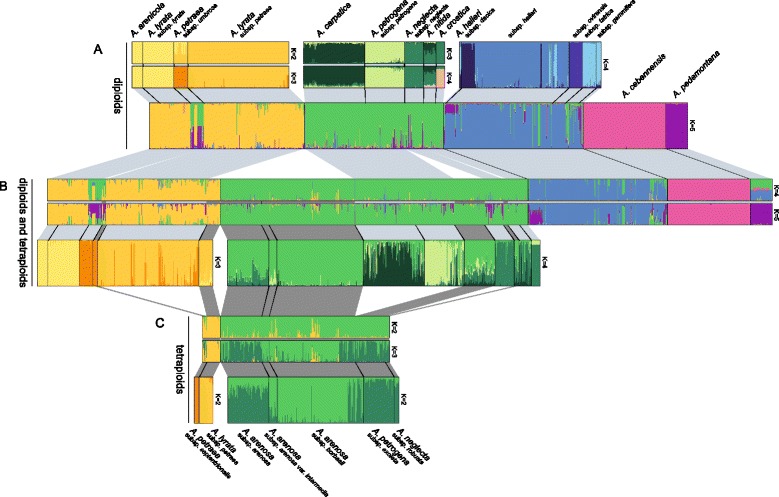
**Genetic assignment analysis of microsatellite data in the genus** ***Arabidopsis.*** Genetic assignment analysis of microsatellite data running STRUCTURE [82,83]. **A)** Total diploid data set separated from the total dataset, **B)** Total dataset comprising all diploids and tetraploids, **C)** Tetraploid dataset. For all datasets and analyses **(A)**, **(B)** and **(C)** significantly recognized groups have been analyzed further individually (groups separated by white spacers: **(A)** upper part, **(B)** lower part, **(C)** lower part. The *A. lyrata* group is highlighted in orange and yellow, *A. arenosa* in green, and *A. halleri* in blue, respectively. The optimal K-values for the various datasets are given with Additional file 4. The total dataset **(B)** upper part has been divided into an analysis with *K* = 4 and *K* = 5.

Gene diversity statistics for microsatellite variation among tetraploid populations was not calculated, but respective data are analyzed and displayed with hierarchical Structure analysis (see Figure 5).

Structure analysis combining diploids and tetraploids recognized five major groups: 1) *A. lyrata*, 2) *A. arenosa* (including *A. croatica*), 3) *A. halleri*, 4) *A. pedemontana* and 5) *A. cebennensis* (Figure 5B, upper part, corresponding Structure-sum analyses provided in Additional file 4). Two of these groups (*A. lyrata* and *A. arenosa*) consist of diploids and tetraploids, and Structure was rerun to analyze these two groups separately (Figure 5B, lower part). In this separate analysis, *A. lyrata* could be split into *K* = 3 populations I) North American *A. lyrata* subsp. *lyrata* and *A. arenicola*, II) diploid *A. umbrosa* and tetraploid *A. septentrionalis*, and III) European *A. lyrata* subsp. *petraea* (irrespective of ploidy level, Figure 5B, lower part).

*A. arenosa*, on the other hand, fell into *K* = 4 populations; all tetraploid *A. arenosa* including tetraploid *A. neglecta* and *A. petrogena* are set apart from the diploid taxa. Vice versa the diploid *A. carpatica* and diploid *A. petrogena* formed distinct groups, respectively. The remaining diploids *A. neglecta*, *A. croatica* and *A. nitida* are combined to form one group. Interestingly, Structure was indifferent to the level of ploidy and the results are total in agreement with the ITS data. However, the initial round of analysis (combining diploids and tetraploids, Figure 5B) produced minor incongruencies such as the occurrence of distinct *A. pedemontana* genetic variation in other species such as *A. umbrosa*. This similarity is immediately eliminated when increasing *K* to the next higher values (data not shown).

When the Structure analysis is strictly confined to each ploidy level separately, the results are more congruent with better resolution (Figure 5A). In the diploid *A. lyrata* dataset (*K* = 2/3), *A. arenicola* was again not separated from *A. lyrata* subsp. *lyrata*, but *A. umbrosa* and *A. lyrata* subsp. *petraea* were still distinguished from each other (Figure 5A, upper part). Note that we report multiple *K*’s here and their corresponding barplots in Figure 5 to reflect the fact that delta *K* was frequently either very similar between two independent runs, or because the less optimal *K* was more biologically meaningful. Both the optimal *K*, and the next-best optimal *K* in the Structure analysis are thus reported.

The diploid *A. arenosa* group was structured with *K* = 3/4, and *A. carpatica*, *A. petrogena* subsp. *petrogena*, *A. neglecta* subsp. *neglecta* and *A. croatica* were significantly recognized. *Arabidopsis nitida* (only few samples analyzed) was less clearly recognized. The five subspecies of *A. halleri* (*K* = 4) were grouped with some genetic clusters that distinguished a) *A. halleri* subsp. *ovirensis*, b) *A. halleri* subsp. *tatrica*/*gemmifera*, and c) a more complex and mixed cluster of *A. halleri* subsp. *halleri* (Figure 5A, upper part). With subsp. *dacica* the results should be interpreted with caution since only three individuals were analyzed. In summary the structure within *A. halleri* subsp. *halleri* is not clear and possibly indicates the need for taxonomic re-evaluation after comprehensive phylogeographic analysis [30,31].

Analysis of the tetraploid dataset resulted in two unambiguously detectable genetic clusters only (Figure 5C, upper part) and distinguished *A. lyrata* from *A. arenosa*. However, it is interesting to see that *A. septentrionalis* carried approximately 50% genetic admixture from the *A. arenosa* genetic cluster. When analyzing tetraploid *A. lyrata* separately (*K* = 2), again *A. septentrionalis* was significantly different from tetraploid *A. lyrata* subsp. *petraea* from Scotland and Austria. In contrast, the tetraploid *A. arenosa* group was structured much less clearly (*K* = 2). At best, two groups could be identified: a) mountainous *A. arenosa* subsp. *borbasii* and high-alpine *A. arenosa* var. *intermedia*, and b) remaining *A. arenosa* subsp. *arenosa*, *A. petrogena* subsp. *exoleta* and *A. neglecta* subsp. *robusta*. It should be noted that neither ITS data nor plastid DNA data differentiated these groups further.

### Genetic diversity is similar in all major groups

Gene diversity and nucleotide diversity is similar among the various groups of taxa (Table 4). Microsatellite gene diversity is highest in *A. lyrata* and *A. arenosa* and significantly lower in *A. halleri*. But this pattern is reverted when considering plastid DNA, where *A. arenosa* shows significantly lower diversity values compared to the *A. lyrata* and *A. halleri* groups. ITS diversity values could be summarized in that *A. lyrata* comprises the most diverse group. Thus, there is some coincidence with wide distribution ranges (*A. lyrata* and *A. halleri*) and high overall genetic variation. However, considering that the *A. arenosa* group has a much smaller total distribution range compared to the others, it is remarkable that levels of genetic diversity are also high. For the two local endemics, *A. cebennensis* and *A. pedemontana,* genetic diversity values are consistently lower.

Mating system affects genetic diversity in the various species and populations. However, detailed information on sporophytic self-incompatibility and mating system is available for *A. lyrata* and *A. halleri* only [50-52]. Both are self-incompatible with few exceptions (e.g. few populations of *A. lyrata* subsp. *lyrata*). Also for the *A. arenosa* lineage there are only reports of a fully self-incompatibility system [29] and there is only one questionable report of a selfing population, so far [53]. Two of the proven allopolyploids (*A. suecica*, *A. kamchatica,* not analyzed herein) are self-compatible [24,54]. For many of the remaining taxa and cytotypes no data were available, and we added our results from many inbreeding experiments at Heidelberg Botanical Garden (2003-2014) to Table 3. Most of these taxa are also self-incompatible, and only for *A. arenicola* was self-compatibility shown, which is well-reflected in lowest number of alleles per locus (Table 3) and gene and nucleotide diversity of any marker system used herein (Table 4). Gene diversity (microsatellites, ITS and cpDNA) and nucleotide diversity (ITS and cpDNA) are for both *A. arenicola* and *A. lyrata* subsp. *lyrata* significantly lower than the respective mean values of the whole lyrata group (t-test: *P* <0.01). The self-incompatible mating system demonstrated for *A. cebennensis* in our cultivation experiments might not fit with its low values of number of alleles per locus (Table 3) or gene diversity (Table 4). However, these low numbers might be also explained simply by its narrow endemic distribution and small population sizes.

### Polyploidy characterizes species groups differently

Mapping of polyploidy levels across the different taxa in our sample reveals that some lineages consist of diploids only (*A. halleri*, *A. cebennensis*, *A. pedemontana*, Table 1). The origins of tetraploid lineages are less clear e.g. tetraploid *A. lyrata* occurs at low frequency in Great Britain and Austria and there is evidence of introgression from tetraploid close relatives such as *A. arenosa* [29]. Distinguishing between a simple doubling of diploid *A. lyrata* genomes within a single ancestral population (autopolyploidy), or the establishment of polyploid lineages as a result of hybridization and genome doubling between two divergent species (*A. lyrata*/*A. arenosa*, allopolyploidy) requires further investigation in this system.

For others such as tetraploid *A. septentrionalis,* no evidence has been presented for a hybrid origin. The most diverse group of taxa with respect to ploidy variation is the tetraploid and diploid lineages within *A. arenosa*. Of the ten listed taxonomic units within *A. arenosa*, five are tetraploids (Table 1 and Additional file 5). As a source of raw material for natural selection to shape novel genes, this genome duplication may well have contributed to genomic instability, leading to genome rearrangement and a driver of speciation in this group.

Only for the very rare *A. umezawana* (from the *A. halleri* lineage) is no chromosome data available, and unfortunately no leaf material was available for microsatellite analysis. Since sequence data (ITS and chloroplast DNA) do not favor any hybrid origin and the various *A. halleri* segregates are exclusively diploid, *A. umezawana* probably also represents a diploid taxon. For *A. croatica* there are diploid and tetraploid chromosome number reports, but the few reports of tetraploids in the field [53] suggest misidentifications as for *A. arenosa* (given the geographic origins of the samples).

## Discussion

We have provided some historical evolutionary context for many of the non-model lineages that comprise the *Arabidopsis* genus. ITS data provided the most robust signature to separate the main evolutionary lineages (Figures 2 and 5): 1) *Arabidopsis thaliana*, 2) *A. cebennensis*, 3) *A. pedemontana*, 4) *A. lyrata* and its segregates/subspecies, 5) *A. arenosa* with numerous different species and cytotypes and *A. croatica* more distinct from the remainder, and 6) *A. halleri* and its subspecies. This summary excludes two hybrid species, namely *Arabidopsis suecica* and *A. kamchatica* “bridging” *A. thaliana*/*A. arenosa* and *A. halleri*/*A. lyrata*, respectively. These taxa will be discussed subsequently, since there is increasing evidence of substantial gene flow over various species and/or ploidy levels [29,48].

### Taxonomy and systematics of *Arabidopsis halleri* and its relatives

Delimitation of *Arabidopsis halleri* is still debated among taxonomists. Up to five subspecies have been recognized [8,9,15,18] though two of these, *A. halleri* subsp. *gemmifera* (Matsum.) O’Kane & Al-Shehbaz and *A. halleri* subsp. *ovirensis* (Wulfen) O’Kane & Al-Shehbaz, are accepted by some authors as separate species, *A. gemmifera* (Matsum.) Kadota and *A. ovirensis* (Wulfen) A. P. Iljinsk., respectively [11,16].

To date, three predominantly central European subspecies were recognized [15]: subsp. *halleri*, subsp. *tatrica* (Pawł.) Kolník, and subsp. *dacica* (Heuff.) Kolník. The third, Asian *A. halleri* subsp. *gemmifera* is geographically separated from the other two subspecies [18]. We did not detect these three subspecies here. *A. halleri* subsp. *gemmifera* formed a cluster with *A. halleri* subsp. *tatrica. Arabidopsis halleri* subsp. *ovirensis* was originally described as endemic to the eastern Austrian high mountain range at Mount Hochobir (Carinthia). Reports from other localities are most likely based on misidentifications (e.g. from Romania and Ukraine). Unique sequence types (ITS and cpDNA) in the populations from Mount Hochobir are in agreement with this narrow endemic distribution [18,22]. Based on microsatellite data, *A. halleri* subsp. *halleri* is characterized by different distinct genetic clusters, which is in congruence with the multiple *A. halleri* gene pools shown earlier [31]: here there were two gene pools with admixture between them, and *Arabidopsis halleri* subsp. *dacica* did not form a separate genetic cluster. Limited taxon sampling prohibits further interpretation. Although subsp. *tatrica* did not show genetic distinctiveness in this study, there is “genetic evidence” for the subspecies *A. halleri* subsp. *tatrica* [31]. Based on the data presented here, we suggest recognizing five subspecies within *A. halleri*: *gemmifera*, *tatrica*, *halleri*, *ovirensis*, and *dacica*, of which *A. halleri* subsp. *ovirensis* is a genetically distinct local endemic taxon and of which *A. halleri* subsp. *tatrica* and subsp. *dacica* need further and detailed phylogeographic analysis. We had limited access to material from *A. umezawana*, but based on *trnLF* and ITS data it is closest to the various subspecies of *A. halleri*.

The evolutionary history of *Arabidopsis halleri* can be summarized as follows: It has previously been shown that all five subspecies are closely related to each other, and that one major center of genetic diversity is located in the eastern Austrian Alps [18,22,31]. The latter [31] explained this center of genetic diversity by secondary contact and admixture of different European gene pools. Similar to the heavy-metal hyper accumulator *N. caerulescens* [55], it was concluded that *A. halleri* metallicolous populations were founded independently from non-metallicolous populations without suffering from founder effects [30]. We think that radiation within *A. halleri* is likely to have occurred during Pleistocene glaciation and deglaciation cycles [22], which also fits with estimates [56] suggesting it to be 335,000 [272,800–438,200] years ago for subsp. *halleri*. Note that this study lacks other subspecies, so a deeper evolutionary split is possible. Furthermore, microsatellite data suggest that *A. halleri* subsp. *gemmifera* may have originated from *A. halleri* subsp. *tatrica* from the Tatra Mountains.

### Systematics of *Arabidopsis arenosa* spp. in relation to resolved gene pools

*A. arenosa* represents a diploid-tetraploid species complex composed of mainly biennial and predominantly outcrossing individuals [53]. The species complex has a distribution range covering most of Eastern Europe and is found in colline to high-alpine habitats exhibiting wide ecological amplitude, spanning from coastal sand dunes to high-alpine screes. Depending on the author, the *A. arenosa* complex comprises several taxa at various taxonomic levels The complex has been treated as one species, *A. arenosa*, with two subspecies of partly overlapping distribution ranges in Central Europe [3]: the tetraploid subsp. *arenosa*, also occurring in northern Europe, growing mainly on siliceous bedrock and sandy soil, and the tetraploid subsp. *borbasii*, growing predominantly on calcareous bedrock in mountainous regions. Diploid *A. neglecta* was described mainly from the Carpathians and rarely from the Alps, where its occurrence is doubtful, since in the Alps this taxon was referred to as *Cardaminopsis arenosa* var. *intermedia* [57]. However, we clearly show that this taxon is closer to tetraploid *A. arenosa* subsp. *borbasii*. Based on morphological and karyological data, several additional (mainly) diploid Carpathian taxa were proposed at the species and subspecies level, and attributed to the genus *Cardaminopsis* [53,58]. Some of these names were never published, however, and kept as “nomina provisoria” (nom. prov.) [59]. Taxonomic concepts in the *A. arenosa* species complex are still strongly debated [18], and we have endeavored to provide clarification here. The lack of resolution for the slower mutating ITS and *trnLF* regions suggests that (recent) radiation within the Pleistocene is plausible for this species complex (the presence of shared ancestry notwithstanding). Our Structure results distinguish mountainous-alpine tetraploid *A. arenosa* subsp. *borbasii* and *A. arenosa* subsp. *arenosa* var. *intermedia* from the remaining tetraploid taxa. Diploid taxa are resolved into *A. neglecta* subsp. *neglecta*, *A. carpatica*, *A. petrogena* subsp. *petrogena*, and *Arabidopsis nitida.* Diploid *A. croatica* is also well separated and shows clear affinities with the *A. arenosa* species group as a whole (see below).

The *A. arenosa* species complex exhibits the highest levels of genetic diversity within the genus. Only *A. lyrata* subsp. *petraea* has comparative values here. In tetraploid *A. arenosa* subsp. *arenosa*/subsp. *borbasii* these levels might be explained by (1) local, periglacial survival, (2) lack of genetic bottlenecks and maintenance of large effective population sizes during postglacial migration into formerly glaciated regions, and (3) gene flow between different taxa and/or ploidy levels [19]. In the cases of *A. carpatica* and *A. petrogena* subsp. *exoleta*, the high levels might be an indicator for past and ongoing speciation within the *A. arenosa* complex in the Western Carpathians [19].

### Taxonomy and systematics of *A. lyrata* and its close relatives

Worldwide, the phylogeography of *A. lyrata* largely reflects its recent introduction by humans. Three biogeographically defined groups have been recognized: Eurasia, the amphi-Pacific region, and North America [27]. However, the most widely used taxonomy recognizes only two corresponding subspecies (*lyrata* and *petraea*), with a third subspecies representing the allopolyploid *A. kamchatica* [8]. Additional Eurasian taxa such as *A. septentrionalis* and *A. umbrosa* have been treated synonymously under *A. lyrata* subsp. *petraea* (*A. arenicola* was at that time treated as a separate taxon) [3]. Our data clearly shows that the North American taxa *A. lyrata* subsp. *lyrata* and *A. arenicola* are close relatives, and that the self-compatible *A. arenicola* probably originated postglacially from *A. lyrata* populations [27].

In accordance with the Panarctic Flora taxonomic concept microsatellites recognized two arctic taxa: *A. petraea* subsp. *umbrosa* and *A. petraea* subsp. *septentrionalis* (Table 1). Both taxa provide a bridge by connecting the European *A. lyrata* subsp. *petraea* with the two North American taxa geographically (and genetically). Remarkably, *A. petraea* subsp. *septentrionalis* represents a tetraploid taxon and given the high genetic similarity of subsp. *umbrosa* with subsp. *septentrionalis,* the latter is most probably an autotetraploid.

### Local endemics and hybrid taxa

**Figure 6:**
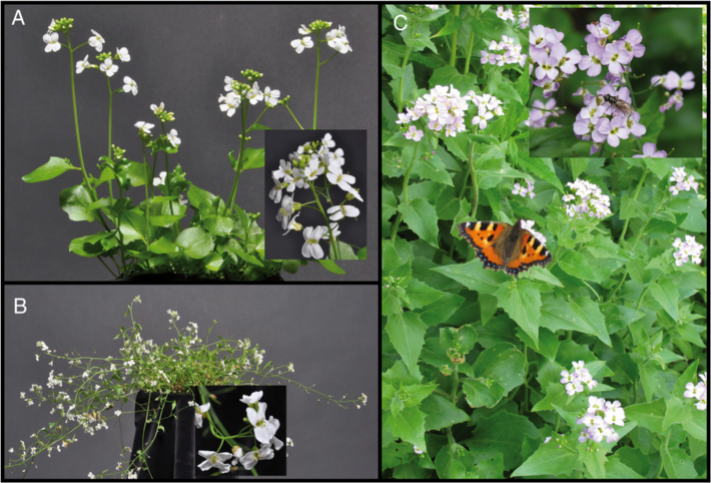
**Growth form of various** ***Arabidopsis*** **species.** Growth form of selected *Arabidopsis* species. **A)** *Arabidopsis pedemontana*, **B)** *Arabidopsis halleri*, **C)** *Arabidopsis cebennensis* (photographs taken by MA Koch (©), U Wagenfeld; Heidelberg).

*A. cebennensis*, *A. pedemontana* and *A. croatica* have distinct highly endemic European distribution ranges (NE Italy, SW France and the Velebit mountains in Croatia, respectively). The species also differ markedly in their ecological preferences and morphology, all of which correlates with the deeper phylogenetic splits inferred among these taxa (Figure 2) and the biogeographic affinity of *A. pedemontana* and *A. cebennensis* to *A. halleri* and of *A. croatica* to *A. arenosa* and *A. lyrata. Arabidopsis pedemontana* and *A. cebennensis* share some traits with *A. halleri,* such as extensive clonal growth, preference for higher moisture, longevity and occurrence at high. Additionally, there is also a striking correlation with phenology, with increasing plant height from *A. halleri*, *A. pedemontana* towards *A. cebennensis* (up to 1.50 m tall), and increased preference of continuously available and cool streaming water in the same sequence of species (Figure 6).

These parallel traits support an evolutionary vicariance scenario in potential refugia west of the main distribution area of *A. halleri*, which itself is distributed along the whole alpine mountain chain. The western and relict occurrence of *A. pedemontana* and *A. cebennensis* may reflect adaptation to refugia during warming phases, i.e. high-and sub-alpine spring habitats with cool streaming water. Our data did not provide enough power for divergence time estimates, but it seems likely that speciation in *A. pedemontana* and *A. cebennensis* occurred during early Pleistocene glaciation and deglaciation cycles.

*A. croatica*, on the other hand, is morphologically and ecologically much closer to diploid taxa of *A. lyrata* and *A. arenosa*. As such, it could be regarded as a derivative of the ancestral gene pool of these respective diploid species (e.g. *A. petraea* subsp. *lyrata*, *A. carpatica*, *A. petrogena*) (Figure 2) [19].

We did not consider in detail here hybrid taxa such as *A. kamchatica* and *A. suecica*. But it is notable that there is increasing evidence of substantial interspecies and interploidal gene flow [48]. It is accepted that *A. kamchatica* has a multiple polytopic origin [27,28], and there is increasing evidence that *A. suecica* does not result from a single hybridization event [60] but rather, multiple events with genetically distinct parents (Polina Novikova, Magnus Nordborg, personal communication), demonstrated and summarized earlier [18]. The first sightings of diploid *A. arenosa* from the Baltic Sea area is now documented (Filip Kolář, Karol Marhold, personal communication), and future genomic analyses will highlight the relationships with putative parental populations of *A. arenosa* and *A. thaliana*. As noted here, *A. petraea* subsp. *septentrionalis* is very likely of hybrid origin, consistent with botanical notes - with limited sampling and one Russian population only - which concluded “… (this population) may have originated from a different refugium probably located more in the East” [36]. Genomic analyses of these endemic and hybrid systems will provide further insight into their evolutionary dynamics.

### Genome size variation in *Arabidopsis*

We did not focus in detail on *A. thaliana*, but as we saw in this study published estimates of nuclear DNA content size in *A. thaliana* also show some genomic variation among wild accessions (1.1 fold difference with a mean 1C value of 0.215 pg) [61]. Absolute values in pg are discussed critically in the same work, and differ largely from other estimates [62,63]. Published results from larger-scale studies were obtained by comparing *A. halleri* and *A. lyrata* while focusing on allopolyploid *A. kamchatica* [64], plants from the latter had a slightly smaller genome size than the sum of its diploid parents. The same study could differentiate genome sizes at the subspecies level, by comparing *A. kamchatica* subsp. *kamchatica* and subsp. *kawasakiana* (larger genome). Data from *A. kamchatica* [64] showed only small differences compared to data from much smaller sample sizes [65] and focusing on *A. lyrata*. Another large-scale study [66] focused on European *A. lyrata* and *A. arenosa* and demonstrated slightly but significantly larger nuclear DNA content in *A. lyrata* compared to *A. arenosa* and its segregates (with both ploidy levels). However, in general there is only a limited number of genome size studies within the genus *Arabidopsis*. Published genome size with the smallest genome size found in *A. arenosa* (1Cx of 0.2 pg) and the largest genome size observed in *A. cebennensis* (1Cx of 0.29 pg) confirm our results [63].

Some discrepancies are apparent among published studies when comparing absolute values of genome sizes either given in pg or in Mbp but this is mostly due to deviations in methodology (e.g. different standards, different fluorescent dyes, sample preparation, diurnal variation within a sample) [64].

### Taxonomic remarks

We do not formally propose new taxonomical combinations but rather highlight some changes (below) which need to be implemented pending completion of more detailed morphological and phylogeographic analyses.

#### *Arabidopsis arenosa* subsp. *arenosa* var. *intermedia*

This taxon is best kept as subsp. of *A. arenosa*, namely *A. arenosa* subsp. *intermedia*. This reflects at best that all tetraploid segregates of the *A. arenosa* group closely belong to each other, but also considers the morphological distinctiveness and local alpine occurrence of *A. arenosa* subsp. *intermedia*.

#### Arabidopsis umezawana

We have no evidence of a hybrid origin (e.g. close affinities to hybrid *A. kamchatica*), but instead convincing evidence that it falls into the *A. halleri* group. Consequently the taxon is at best treated as a subspecies, namely *A. halleri* subsp. *umezawana*.

#### Arabidopsis arenicola

This taxon is closest related to North American *A. lyrata* subsp. *lyrata*. Consequently, it should be treated as subspecies under a North American *A. lyrata*, namely *A. lyrata* subsp. *arenicola*. Morphological differences are weak: Compared to *A. lyrata* subsp. *lyrata* fruits are terete or only slightly flattened, and cotyledons are incumbent [67].

#### *A. lyrata* subsp*. petraea*

*Arabidopsis petraea* subsp. *septentrionalis* and *A. petraea* subsp. *umbrosa* have already been described and characterized within the Pan Arctic flora project as subspecies within *A. petraea*. There are two different options to solve this taxonomic/phylogenetic incongruence. 1) European *A. lyrata* subsp. *petraea* is treated as *A. petraea* subsp. *petraea*, thereby taking geographical and genetic affinities into account and not changing taxonomy of subsp. *septentrionalis* and *umbrosa*. 2) Treating members of the *A. lyrata* group on subspecies level and establishing the new combinations *A. lyrata* subsp. *septentrionalis* and *A. lyrata* subsp. *umbrosa*. We prefer the second option, since this would minimize future confusion and misuse of species names [21]. Clearly more and detailed studies of these two taxa are needed. From the herein presented microsatellite analysis it could be hypothesized that the *A. halleri* genome is also introgressed into both species. The Structure analysis shows some affinities with the purple genetic cluster and linking in particular subsp. *septentrionalis* and *umbrosa* with some populations of *A. halleri* and *A. pedemontana* (Figure 5B, upper part; Figure 5A, lower part), but see comments given above with *A. pedemontana*.

## Conclusion

We characterized in detail the three main *Arabidopsis* evolutionary lineages: *A. halleri*, *A. lyrata* and *A. arenosa*, including their respective subspecies in an attempt to present a genus-wide overview on genetic variation and taxon delimitation. The relationship among these three lineages is not completely certain due to the power of resolution across the assays used here, but there is some tendency that the *lyrata* lineage is more closely related to *arenosa* than to *halleri*, consistent with being sister taxa. Three additional well-defined endemic species, *A. pedemontana*, *A. cebennensis* and *A. croatica* do form separate evolutionary lineages, with the latter (*croatica*) most likely positioned at the base of the *A. arenosa* lineage. The other two endemics are distantly related to any other lineage, but ecologically and morphologically closer to *A. halleri*. Aside from these evolutionary lineages, there is a need to characterize some taxa in much more detail, such as the arctic taxa of *A. lyrata* and members of the *A. arenosa* species aggregate. One other conclusion which stems from the extensive chloroplast haplotype sharing observed among all major evolutionary lineages is the need to qualify and quantify the extent of gene flow within the entire genus.

## Methods

### Plant material and general sampling strategy

This study was designed to incorporate as much existing data as possible in order to provide a comprehensive perspective on taxon sampling as well as their geographic (spatial) distribution. The internal transcribed spacers (ITS1 and ITS2) separating the small and large rRNA subunits and the plastid *trn*L intron including the adjacent *trn*L-F intergenic spacer (hereafter called *trn*LF region). A single individual of respective accessions rather than population-based sampling is common however from these earlier publications. Consequently, many new species accessions and population level sampling have now been added to the existing sample pool. All new individuals have been genotyped using microsatellites using methods established and optimized for the characterization of an *Arabidopsis* hybrid zone [29].

The different sampling levels (populations versus individual accessions) is also the main reason why ITS and *trn*LF data are visualized as trees and/or networks (phylogenetics), and microsatellite data were subject to population based algorithms. World-wide sampling localities are provided in Additional file 5 (including GenBank accession numbers) and illustrated in Figure 1. In brief, we sampled 2909 individuals from 813 populations/accessions representing all taxa and cytotypes of the genus *Arabidopsis* (see also Table 1). For individual marker sets the sampling is as follows: ITS, 1120 individuals/524 accessions; *trn*LF, 1777 individuals/632 accessions; microsatellites, 1345 individuals/222 accessions; and cytogenetic analysis, 221 accessions. Note that not all sample material was of sufficient quantity or quality for PCR (material included: voucher, wild, living collections). Information on ploidy level (Table 1) is either based on chromosome counts, genome size measurements or indirectly by the numbers of alleles per locus (based on microsatellite genotyping) (see [19,29] for cytological methods). Unambiguous detection of polyploids using microsatellite genotyping is, of course, only possible if more than two alleles are present at a given locus.

### DNA isolation, amplification, and sequencing

Total DNA was obtained from dried leaf material and extracted according to a CTAB protocol [68] with the following modifications: 50–75 mg of dry leaf tissue were ground in 2 ml tubes using a Retsch swing mill (MM 200), 2 units of RNase A per extraction were added to the isolation buffer, and the DNA pellets were washed twice with 70% ethanol. DNA was dissolved in 50 μl TE-buffer for storage and diluted 1:3 in TE-buffer before use.

For the cpDNA markers *trnL* intron and *trnL/F* intergenic spacer (*trnL/F-IGS*), primers and PCR cycling scheme followed the protocol of [27,69], using a PTC200 (MJ Research, Waltham, USA) thermal cycler. The PCR reaction volume of 50 μl contained 1x PCR buffer (10 mM TRIS/50 mM KCl buffer, pH 8.0), 3 mM MgCl_2_, 0.4 μM of each primer, 0.2 mM of each dNTP, 1 U Taq DNA polymerase (Amersham Biosciences, Chalfont St Giles, England), and approximately 5 ng of template DNA. Amplified sequences of *trnL/F-IGS* included the complete *trnL/F-IGS* and the first 18 bases of the *trnF* gene. Amplification of the ITS region was performed according to [70]. PCR reaction conditions were the same as for the two cpDNA markers described above, and PCR cycling scheme was 5 min at 95°C, 35 cycles of 1 min at 95°C, 1 min at 48°C, and 1 min at 72°C, 10 min extension at 72°C, and a final hold at 4°C. PCR products spanned the entire ITS1, 5.8S, and ITS2 region.

Before sequencing PCR products were checked for length and concentrations on 1.5% agarose gels and purified with the NucleoFast Kit (Macherey-Nagel, Düren, Germany). The sequencing was performed by GATC GmbH (Konstanz, Germany) and Eurofins MWG Operon (Ebersberg, Germany). Additionally, cycle-sequencing was performed on the MegaBase500 system using the DYEnamic ET Terminator Cycle Sequencing Kit (Amersham Biosciences, Chalfont St Giles, England).

### Microsatellite amplification and allele detection

Microsatellites were chosen from previous studies of *A. lyrata* [29,71]. The allopolyploid *A. kamchatica*, *A. suecica* and introgressed tetraploid hybrids of *A. lyrata* subsp. *petraea* and *A. arenosa* were excluded from this analysis. Selection criteria, PCR and genotyping conditions are provided in detail together with a list of the seven SSRs finally chosen for the analyses in our previous contribution [29]. Scoring of fragment sizes and fluorescence intensity/peak heights (in tetraploids) was automatically performed with GeneMarker version 1.95 (SoftGenetics, State College PH, USA) using respective panels for each locus with subsequent manual checking of each sample. Allele frequencies within tetraploid individuals could unambiguously be assigned manually for the majority of individuals, based on the fluorescence intensity of the fragment peaks [29].

### Estimation of nuclear DNA content

Nuclear DNA content was determined using flow cytometry following a simplified two-step protocol [72]. Approximately 10 mm^2^ of fresh leaf tissue (or one fresh petal) from each plant was chopped together with an appropriate volume of the internal reference standard (*Solanum pseudocapsicum*, 2C = 2.59 pg, [73]; an identical individual was used for all measurements) using a razor blade in a Petri-dish containing 0.5 mL of ice-cold Otto I buffer (0.1 M citric acid, 0.5% Tween 20). The suspension was filtered through a 42-μm nylon mesh and incubated for 10 min at room temperature. Isolated nuclei were stained with 1 mL of Otto II buffer (0.4 M Na_2_HPO_4_.12H_2_O) supplemented with propidium iodide and RNase (both in concentration 50 μg mL^-1^), and β-mercaptoethanol in concentration 2 μg mL^-1^. After a few minutes, the relative fluorescence intensity of 5000 particles was recorded using flow cytometer CyFlow SL (Partec GmbH, Germany) equipped with green (532 nm) solid state laser. We applied the following stringent criteria in order to get precise and stable flow cytometric results: (i) only analyses with the coefficient of variation of the sample peak below 3% were taken into account (ii) each sample was measured at least three times on different days to minimize potential random instrumental drift [74], and (iii) the between-day variation was defined to not exceed the 3% threshold; otherwise the most remote value was discarded and the sample was re-analyzed. The histograms were evaluated with FloMax FCS 2.0 program (Partec GmbH, Germany). Differences in homoploid nuclear DNA contents among major gene pools (separately for diploid and tetraploid accessions) were analyzed by one-way ANOVA with TukeyHSD post-hoc comparisons in R v.2.15.2 [75]. The dataset comparing relative genome sizes of taxonomic groups was generated in Prague. A second dataset was generated in Heidelberg to provide some estimates on absolute genome sizes. The second dataset incorporated different standards (*Solanum lycopersicum* cv. Stupicke, 0.98 pg/1C; and *Raphanus sativus* cv. Saxa, 0.55 pg/1C) [76] because of comparing to, and integrating into datasets from all over the Brassicaceae. Respective data are deposited in *BrassiBase* [20,77]. The two datasets were not merged afterwards and kept separate, because accessions analyzed and standards used were different (as explained above).

### ITS and *trn*LF DNA sequence delimitation

Plastidic *trn*LF sequences were defined as haplotypes and suprahaplotypes following previous studies [18,22,26,29]: Haplotypes are characterized by multiple *trn*F pseudogenes in the 3′-region of the *trn*LF*-IGS* close to the functional *trn*F gene [26,78,79]. When defining respective *trn*LF suprahaplotypes, we excluded the pseudogene-rich region and thereby merged sets of haplotypes into suprahaplotypes. The *trn*F pseudogenes evolve with a mutation rate 10 × higher than single nucleotide polymorphisms, which makes them non-applicable for phylogenetic reconstruction at the species level [26,80,81]. In summary, haplotypes belonging to one suprahaplotype share the same base order throughout the whole sequence except for the pseudogene-rich region, where they vary in both length and base content. Suprahaplotypes differ from each other only by single point mutations and/or indels. Newly defined *trn*LF haplotypes were assigned to GenBank [LN610052-LN610063/LN610032-LN610051)] (Additional files 3 and 5). ITS sequences were obtained from direct sequencing of PCR products and defined as previously [18,22,26,29]. A few minor corrections of past ITS type numbering had to be conducted, and codes are indicated in Additional file 5 with new assignments to GenBank [LN610064-610098].

### Network, phylogenetic analysis and genetic diversity statistics

Network analyses and genetic diversity statistics were exclusively performed using the *trn*LF suprahaplotypes, as the pseudogene-rich region is not applicable for phylogenetic reconstruction at the species level [26]. The alignment of the cpDNA sequences was manually made with subsequent adjustment in PhyDE version 0.9971 [82]. The network was constructed using TCS version 1.21 [83] and the statistical parsimony algorithm [84]. Gaps (except polyT stretches) were coded as single additional binary characters. Reliability of certain connections, especially if multiple and internal connections occurred within the network, was tested by analyzing the respective alignment with maximum likelihood-based tree construction methods [85]. Only those connections showing up in both types of analyses were retained. Any unsupported connections are indicated with dashed lines in the respective figure. DNA sequence information from *A. thaliana* was used to set the root.

ITS sequences were also aligned manually with subsequent adjustment in PhyDE version 0.9971 [82]. Maximum parsimony analysis was performed running PAUP 4.0b10 [86] and using *A. thaliana* as an outgroup. The parsimony heuristic search was performed with the following settings: gaps were treated as missing data (using the gap-based coded 0/1-matrix), multi-state taxa were interpreted as uncertainty; tree construction was via stepwise addition; tree-bisection-reconnection (TBR) was implemented via the branch-swapping algorithm; MaxTrees limit was set to 10,000; and the MulTrees option was selected (saving all minimal trees found during branch swapping). For bootstrapping, 1000 replicates with a tree maximum of 500 retained trees were run. The resulting phylogenetic hypothesis was used to manually place the root in a reliable way with the subsequently performed network analysis (SplitsTree 4.13.1; [87]). For the network analysis *A. thaliana* was removed from the dataset to increase resolution of internal splits (removing homoplastic characters).

Genetic diversity statistics were performed with Arlequin version 3.5.1.3 [88] and Nei’s genetic diversity and gene diversity was calculated accordingly [89]. Allopolyploids (*Arabidopsis kamchatica, A. suecica,* introgressed *A. lyrata* subsp. *petraea*) and individuals which could only be assigned to a lineage but not to lower taxonomic units were excluded from the analyses.

### Genotyping of microsatellite alleles and genetic assignment tests

We obtained comparatively full datasets for diploid and tetraploid microsatellite allele scoring (Additional file 6). Microsatellite genotypes were analyzed using Structure 2.3.4 [90,91], with ten replicate runs for each *K*-value, and a burn-in period of 1 × 10^5^ and 2 × 10^5^ iterations. The options ‘admixture model’ was used in combination with ‘uncorrelated allele frequencies’. The estimation of the optimal *K* number of populations (ranging from 1 to 10) was calculated using the R-script Structure-sum [92], which compares the posterior probabilities of the runs [93], the similarity coefficient between the runs, and delta *K* as defined by [94]. In the visualization of Evanno’s delta *K*, a peak had to appear in the optimal fitting model with consistent results over multiple runs [92,94]. Input files for CLUMPP were generated with STRUCTURE HARVESTER [95], alignments of replicate runs were conducted in CLUMPP [96] and the mean of 10 runs was visualized [97]. Note that for some of the more complicated groupings (e.g. diploids with all species accessions) the variance between independent runs for *K* with the highest delta *K* (optimal *K* according to the method of Evanno [94]) was high. In these cases we turned to the variance for guidance concerning the correct *K*, and choose *K* with the lowest variability across runs. At all times we aimed for the smallest value of *K* that captured most of the structure in the data with a clear biological interpretation for individual assignments.

To overcome conceptual restrictions in combining diploid and tetraploid data we conducted three separate analyses: I) on the whole ploidy dataset, this combined diploids and tetraploids where diploid alleles were doubled to mimic tetraploid data; II) diploids only; and III) tetraploids only. Following these three analyses Structure was again run on the subsets of accessions detected by analysis I to III.) The whole dataset (I) comprised 24 taxa and 1345 individuals and was subsequently split into two separate runs. The diploid dataset (II) comprised 17 taxa and 998 individuals and was subsequently split into three separate runs. The tetraploid dataset (III) comprised seven taxa and 347 individuals and was subsequently split into two separate runs. For those subsets we also tested for optimal *K*-values. LocPriors were set with split datasets to optimize search strategies using taxon labels. Two aspects have to be considered regarding the Structure analyses: 1) We excluded *a priori* any known hybrid taxon from the analyses (e.g. *A. suecica*, *A. kamchatica*, *A. lyrata* from the eastern Austrian Forealps and the Wachau in Austria; for details see: [27,29,48], and 2) almost all taxa are obligate outcrossers (known self-compatible exceptions are the few populations of *A. lyrata* in the Great Lakes region of eastern North America: [33,51]; *A. kamchatica* and *A. kamchatica* subsp. *kawasakiana* from Japan: [54,98]; *A. suecica* [99]; *A. arenicola*, this study). It can also be assumed that *A. kamchatica* is self-compatible. Genetic diversity statistics were performed with Arlequin 3.5.1.3 [88] for diploid taxa.

## Availability of supporting data

The data sets supporting the results of this article are available online. A complete documentation of the new sequences generated for this study, including GenBank accession numbers, is available from Additional file 5. Further, data files (accession list; ITS alignment; microsatellite dataset) are accessible with the Dryad data repository under doi:10.5061/dryad.497sg.

## Additional files

Additional file 1:
**ITS alignment. **Fasta-file of ITS types (see Additional file 5 for details). Additional file 2:
**CpDNA alignment.** Fasta-file of *trn*LF suprahaplotypes (see Additional file 5 for details). Additional file 3:
***trn*** **LF haplotype distribution among taxa.**
Additional file 4:
**Delta K runs.**
Additional file 5:
**Accession list with detailed information on origin, DNA data, cytogenetic data and inferences and haplotype definitions.**
Additional file 6:
**Microsatellite data.**
