# Supplementary material for: Taming the wild: resolving the gene pools of non-model Arabidopsis lineages
Source: BMC Evol Biol. 2014 Oct 27;14:224. doi: 10.1186/s12862-014-0224-x (PMC4216345; doi:10.1186/s12862-014-0224-x)

Corresponding to Fig. 5 A, upper part

Lyrata group, diploids, LocPrior

288 individuals, 72 accessions

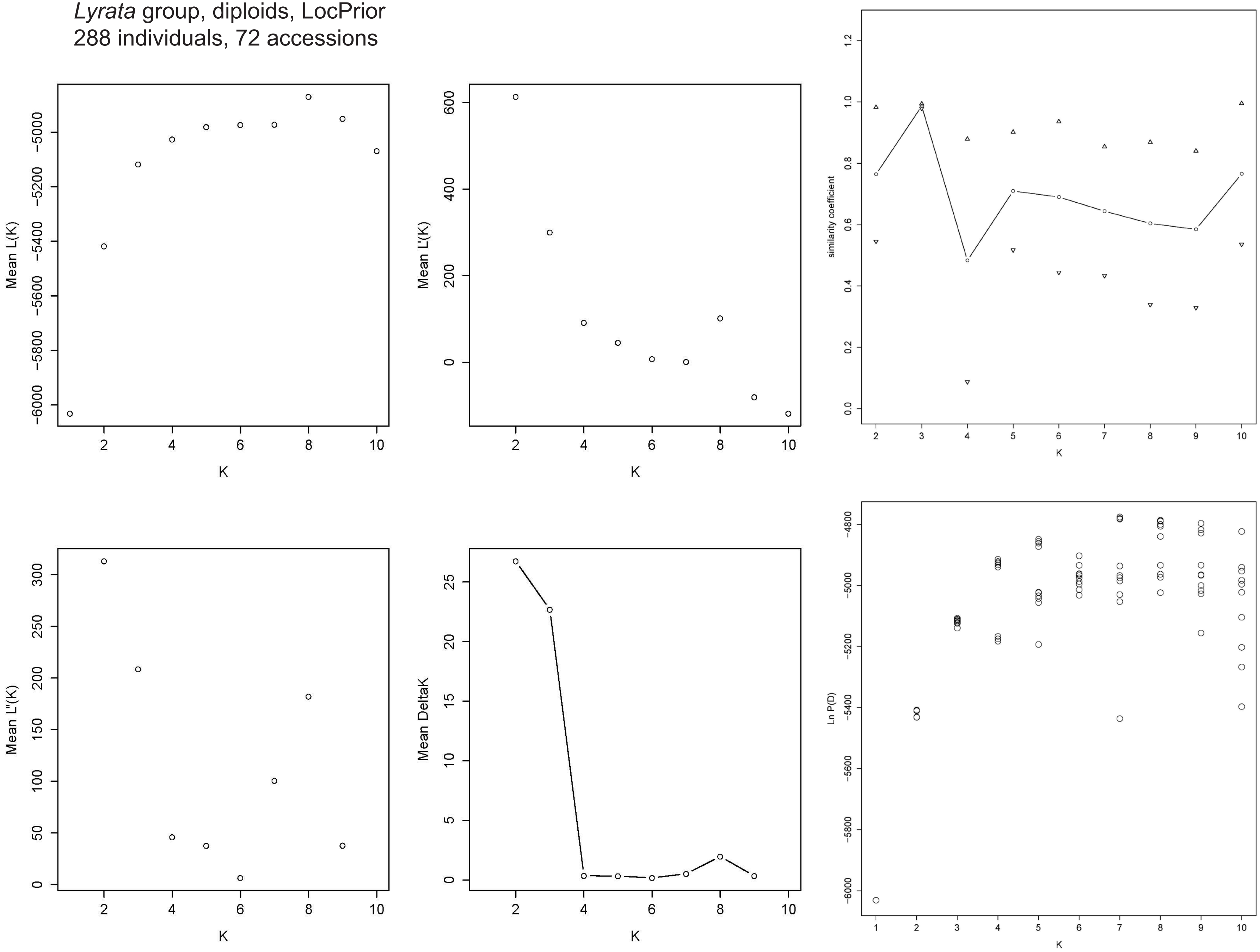

Corresponding to Fig. 5 A, upper part
Arenosa group, diploids, LocPrior
258 individuals, 28 accessions

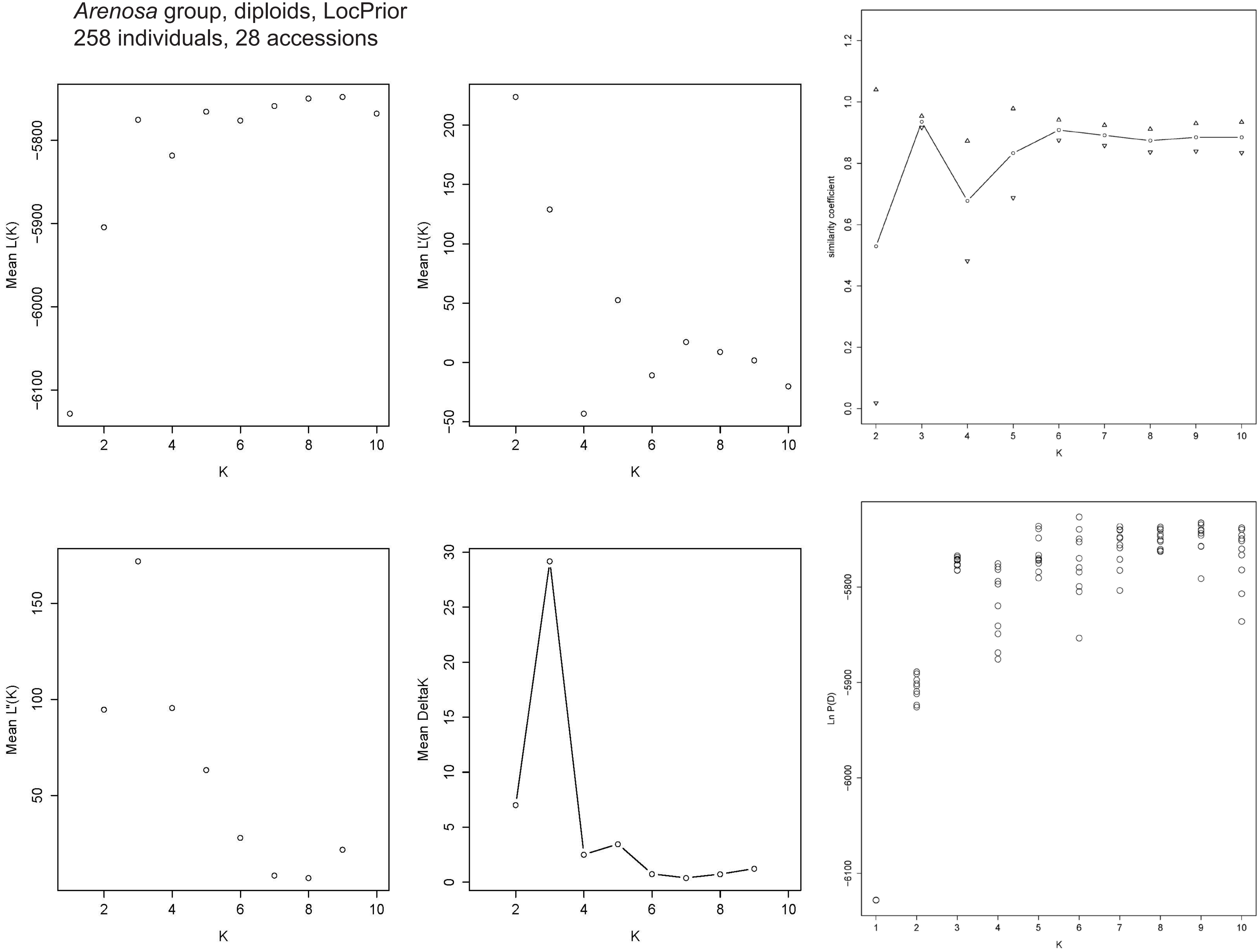

Corresponding to Fig. 5 A, upper part
Halleri group, diploids, LocPrior
259 individuals, 38 accessions

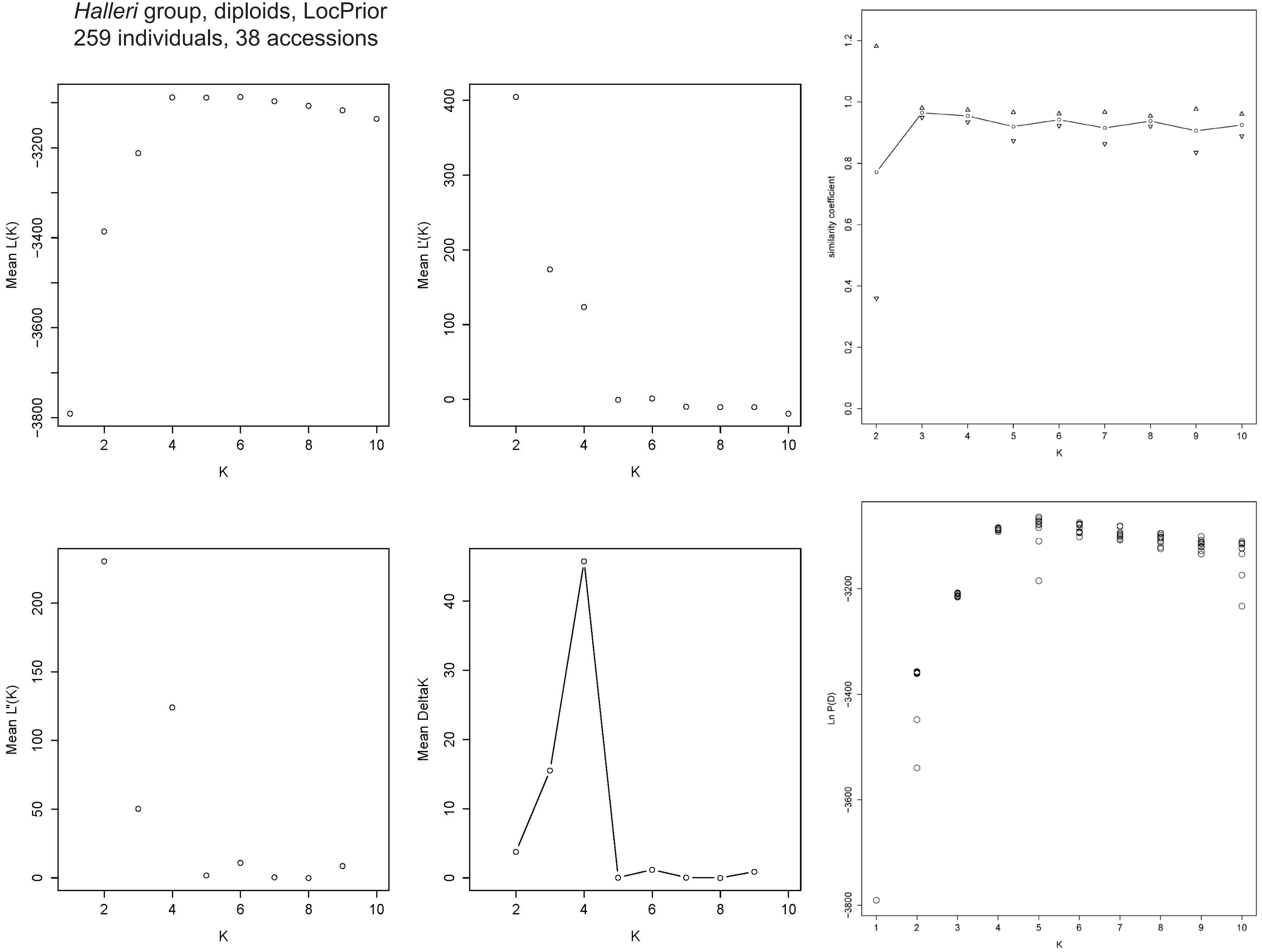

Corresponding to Fig. 5 A, lower part

Complete dataset, diploids, no LocPrior

998 individuals, 158 accessions

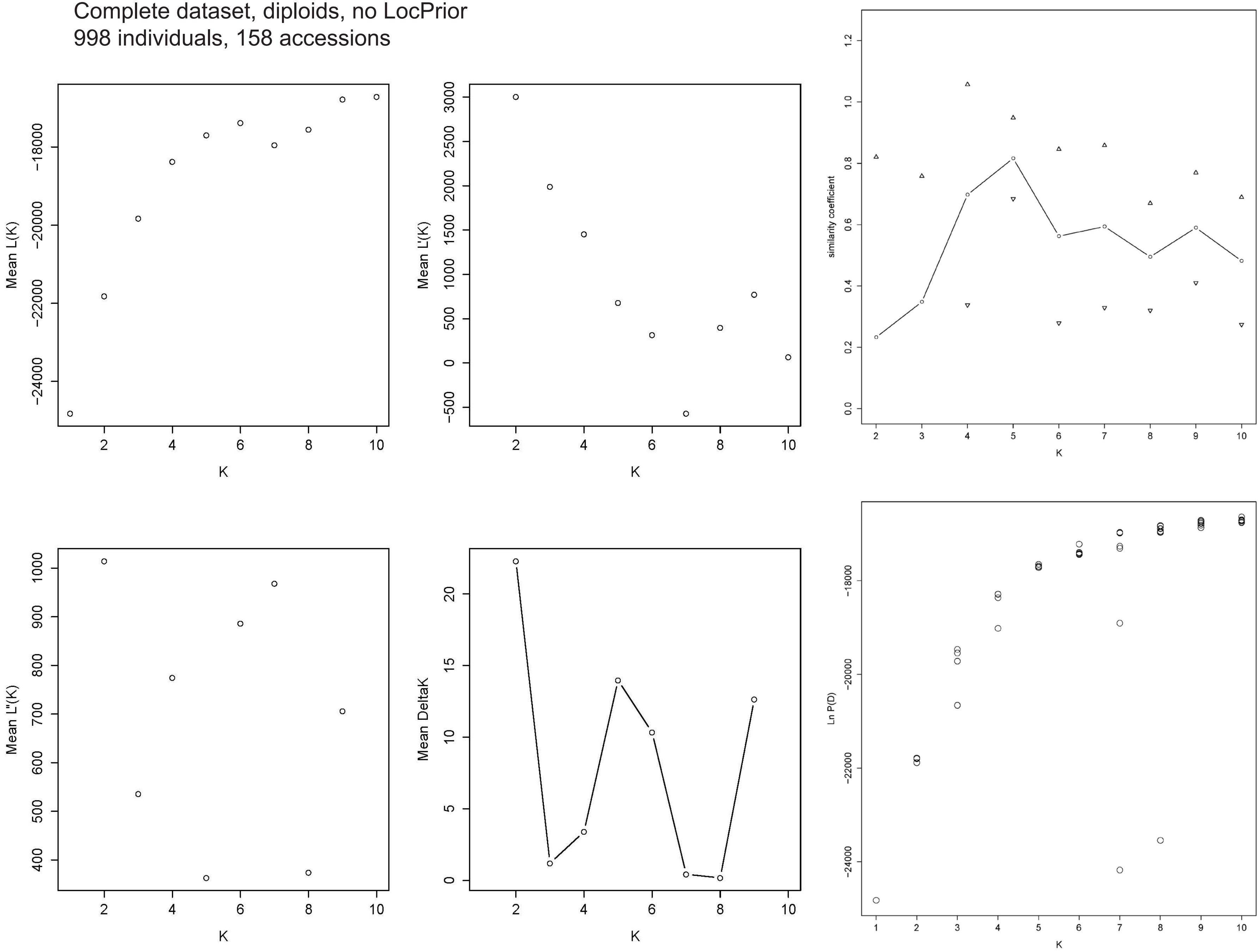

Corresponding to Fig. 5 B, upper part

Complete dataset, diploids and tetraploids, no LocPrior

1345 individuals, 222 accessions

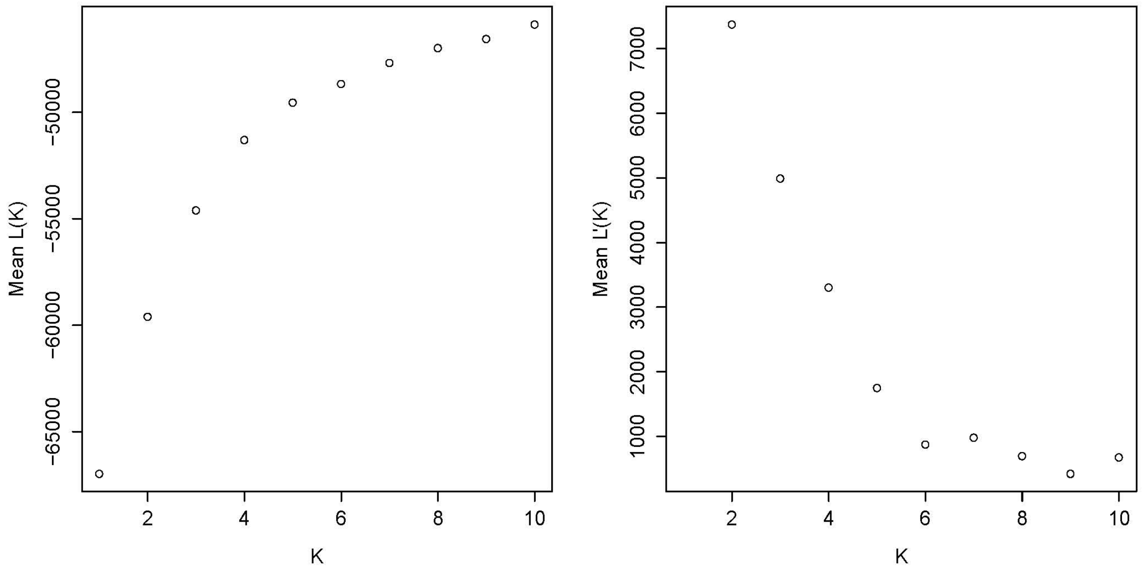

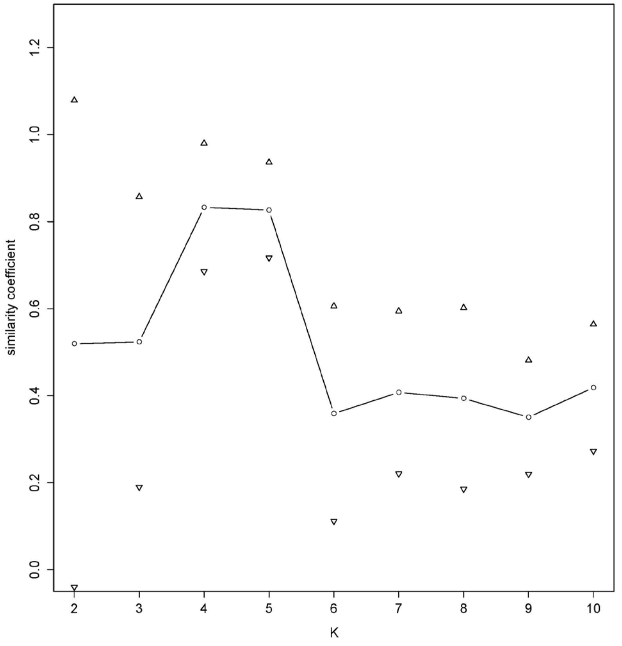

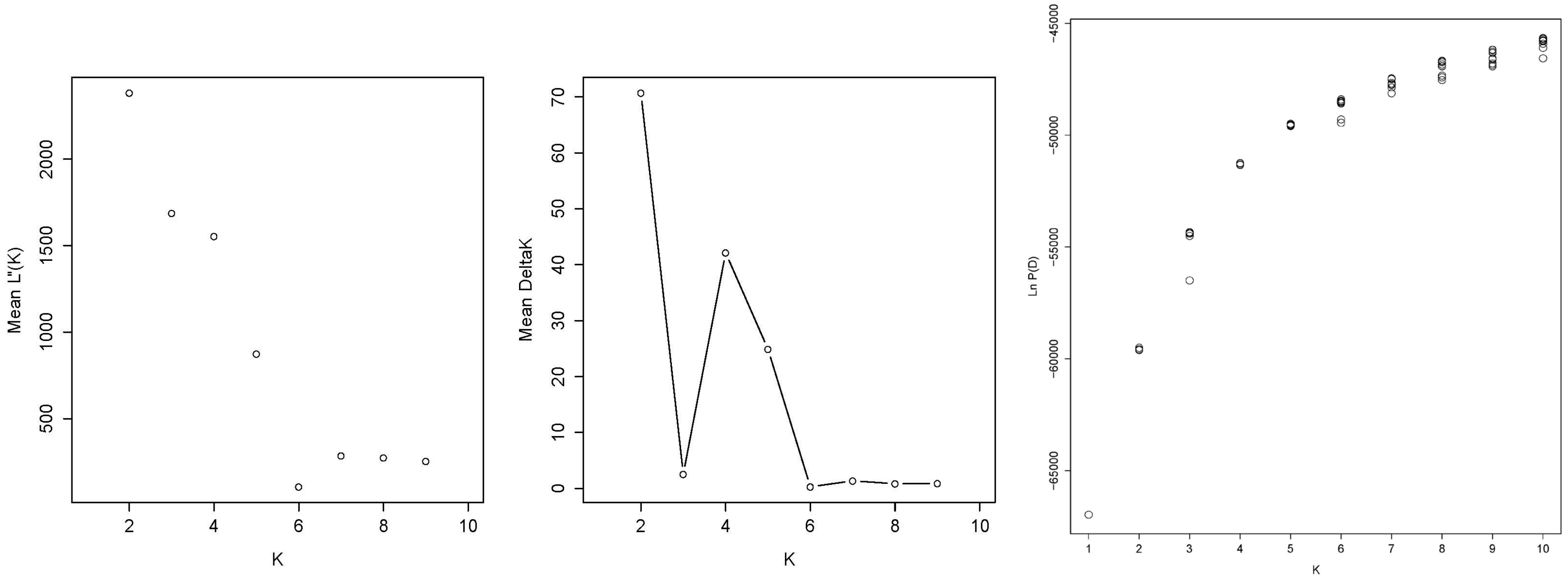

Corresponding to Fig. 5 B, lower part

Lyrata group, diploids and tetraploids, LocPrior

321 individuals, 85 accessions

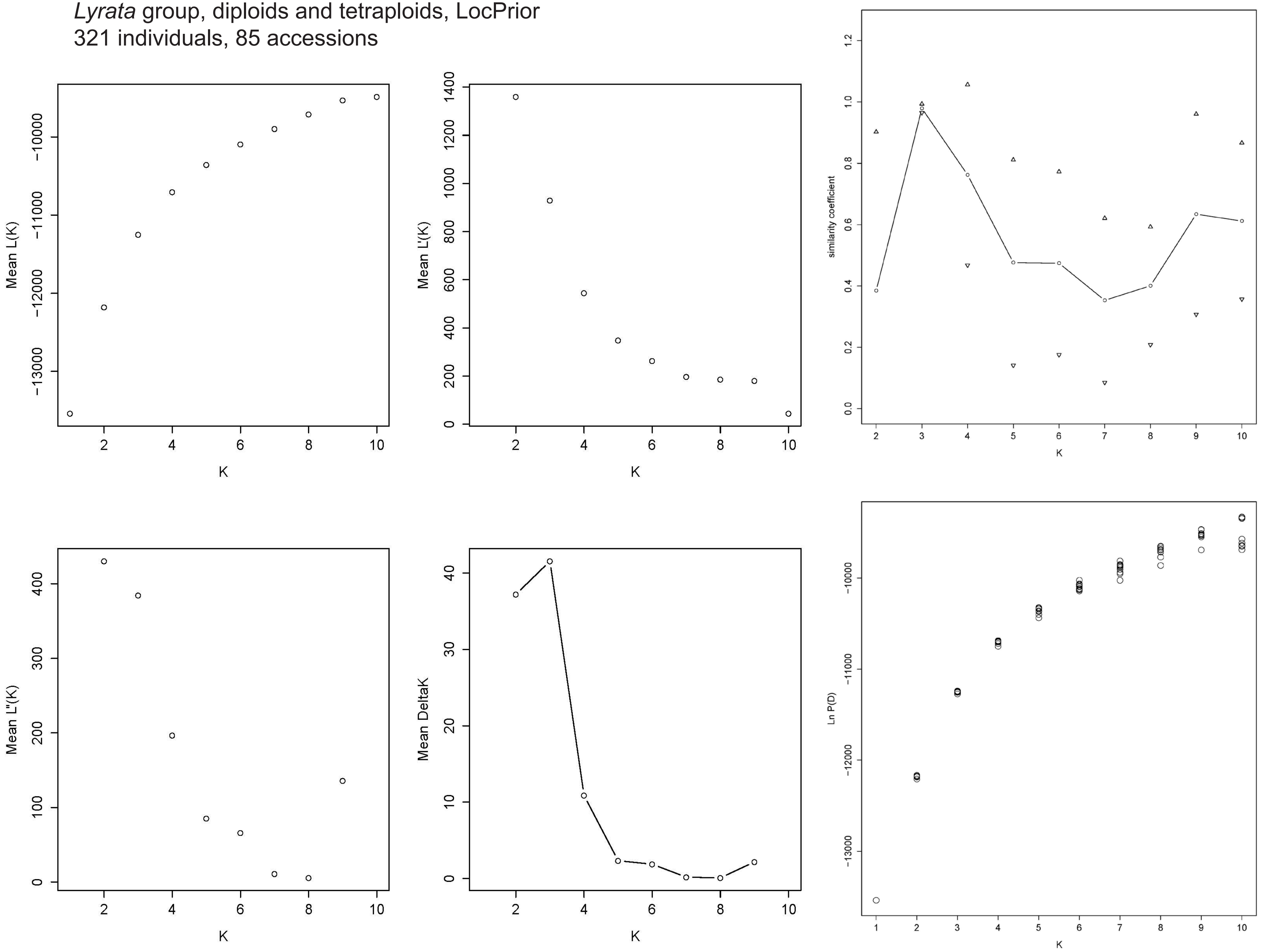

Corresponding to Fig. 5 B, lower part

Arenosa group, diploids and tetraploids, LocPrior

572 individuals, 79 accessions

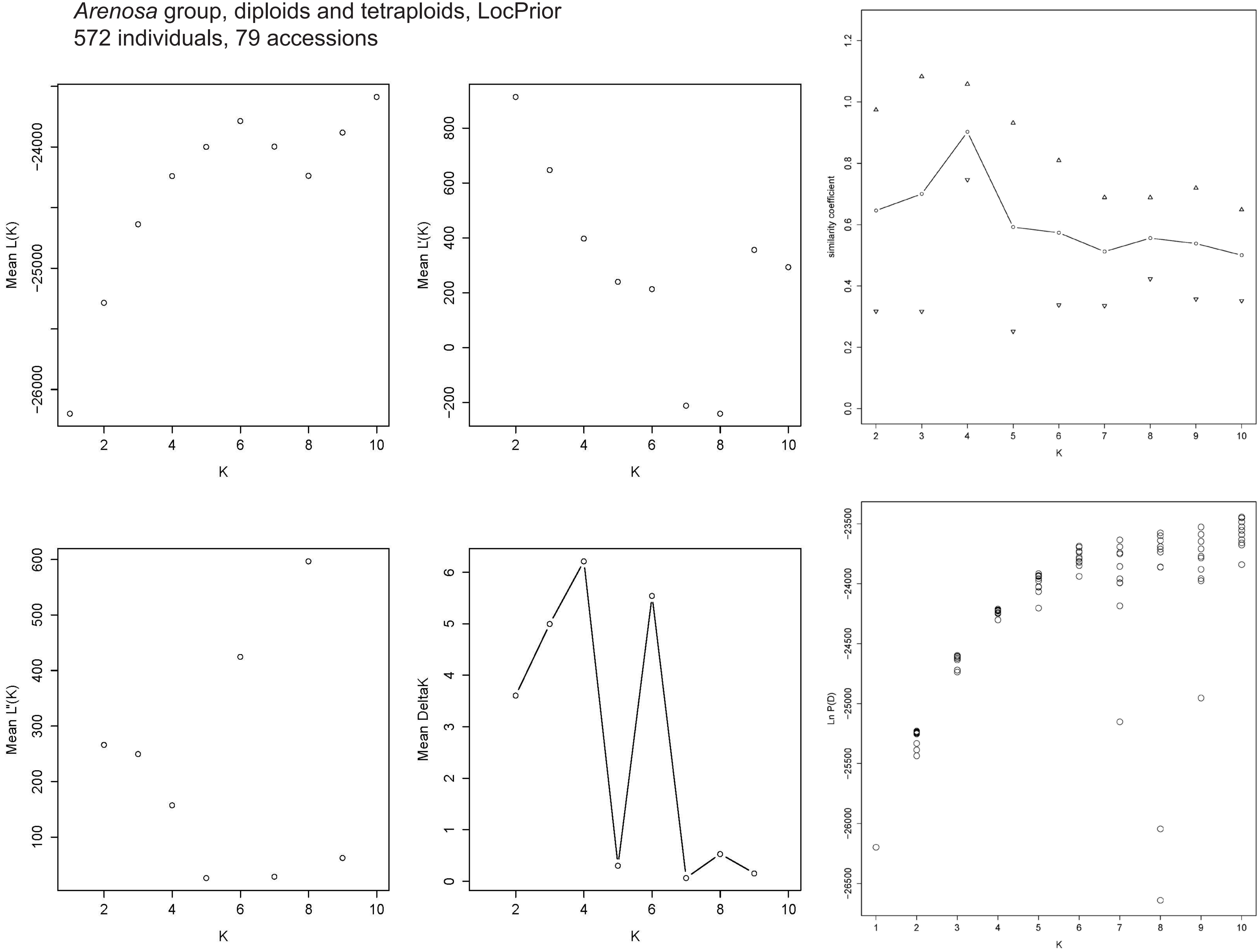

Corresponding to Fig. 5 C, upper part

Complete dataset, tetraploids, no LocPrior

347 individuals, 64 accessions

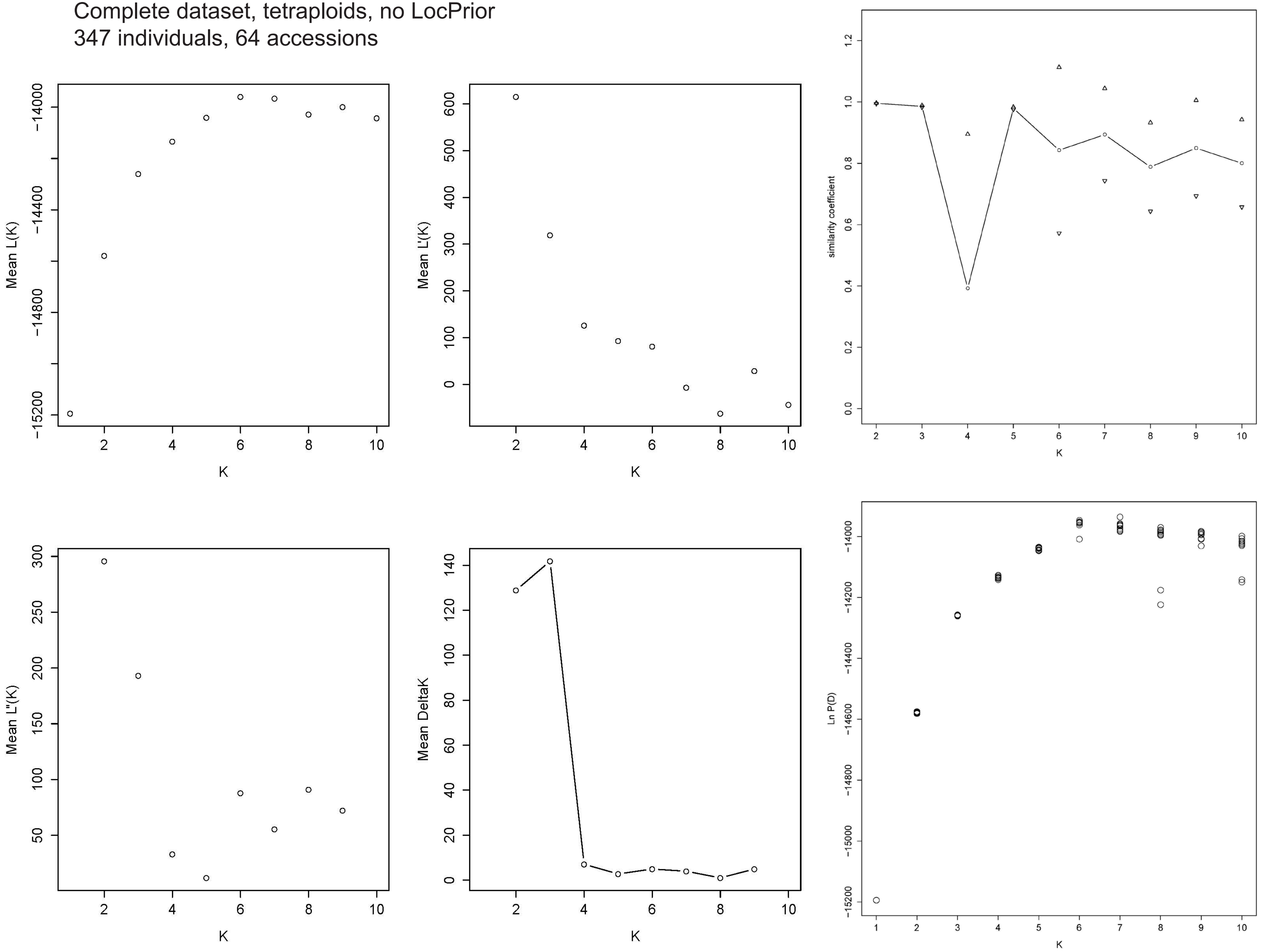

Corresponding to Fig. 5 C, lower part
Lyrata group, tetraploids, LocPrior
33 individuals, 13 accessions

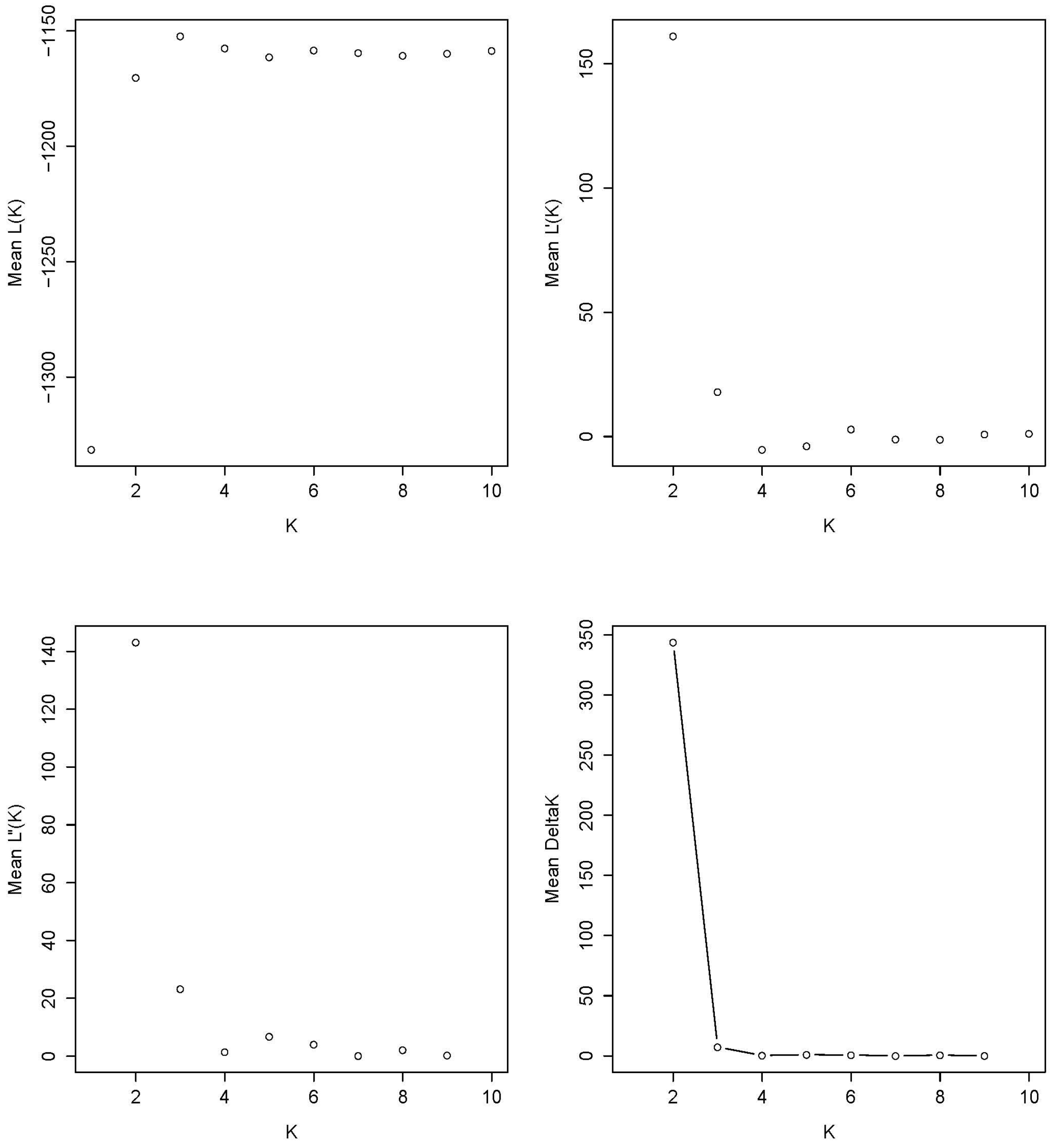

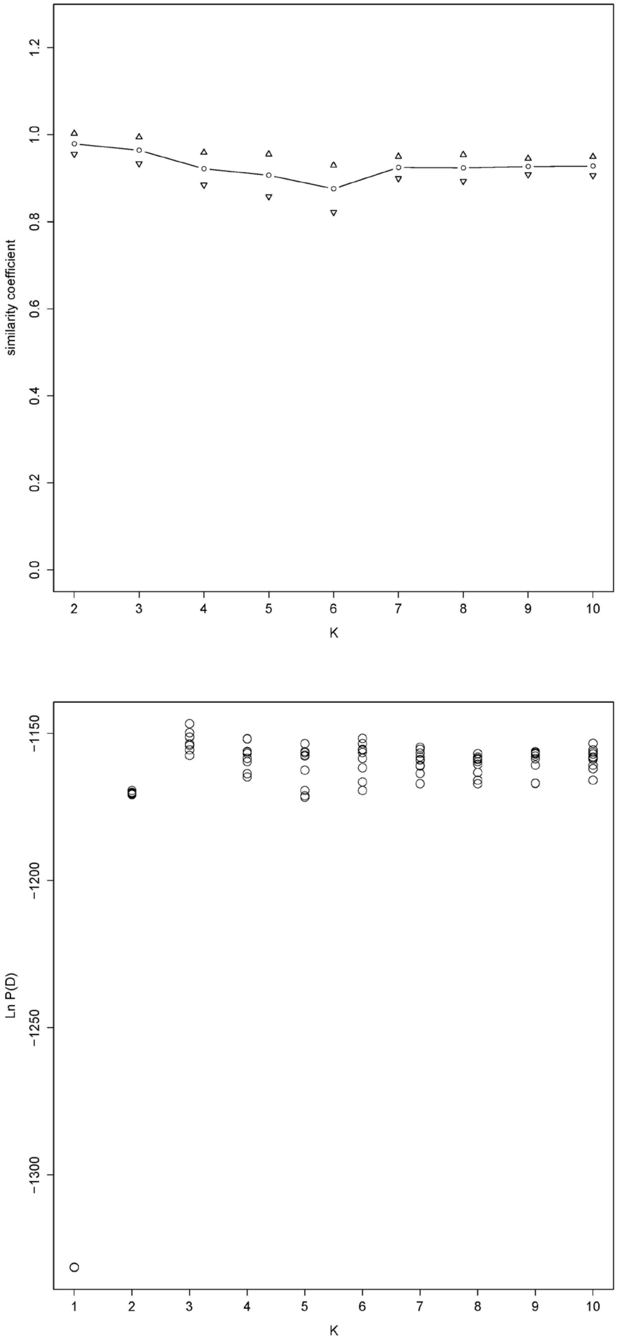

Corresponding to Fig. 5 C, lower part
Arenosa group, tetraploids, LocPrior
314 individuals, 51 accessions

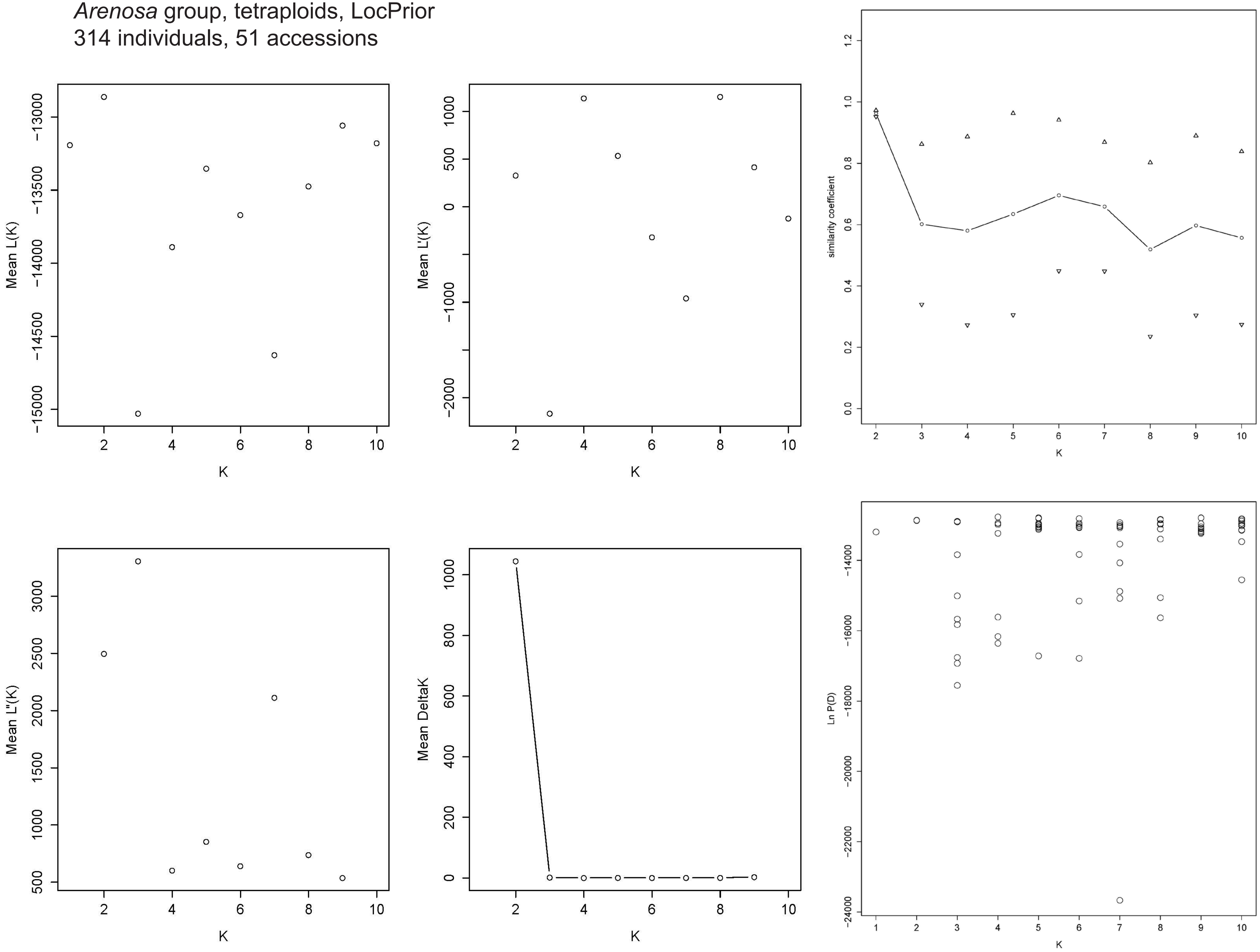

Supplement: Additional file 4: — Delta K runs. [file 12862_2014_224_MOESM4_ESM.pdf]
